# Pan-Cancer Analysis of *PAPPA* Gene Reveals Tumor-Specific Prognostic Effects

**DOI:** 10.3390/biology15060460

**Published:** 2026-03-12

**Authors:** Samah Mutasim Alfadul, Khalid Omama, Alisa Y. Potapova, Pavel A. Ivanov-Rostovtsev, Maryam Fanian, Reem Mubarak, Hind Ahmed Gasimelseed, Minas M. Balla, Amani M. A. Bakhiet, Khalid Berma, Mohamed Alfaki, Maria V. Babak

**Affiliations:** 1 Drug Discovery Lab, Department of Chemistry, City University of Hong Kong, 83 Tat Chee Avenue, Hong Kong SAR 999077, China; salfadul2-c@my.cityu.edu.hk (S.M.A.); a.potapova@cityu.edu.hk (A.Y.P.); p.ivanov-rostovtsev@cityu.edu.hk (P.A.I.-R.); mfanian.88@gmail.com (M.F.); 2 Department of Biology and Biochemistry, Faculty of Sciences I, Lebanese University, Old Saida Rd, Hadath, Beirut 1100, Lebanon; khalidomama2001@gmail.com; 3 Faculty of Pharmacy, International University of Africa, Madani St., Khartoum P.O. Box 2469, Sudan; reemmubarak35@gmail.com; 4 Department of Immunology and Biotechnology, Tropical Medicine Research Institute, National Center for Research, El Mek Nimir Avenue, Khartoum 11111, Sudan; hindahmed43@gmail.com; 5 Faculty of Sciences, University of Gezira, University Avenue, Wad Madani 21111, Sudan; minasmballa@gmail.com; 6 College of Science, Hafr Al-Batin University, King Abdulaziz Rd, Hafar Al-Batin 31991, Saudi Arabia; ambakhiet@uhb.edu.sa; 7 Department of Biotechnology, Faculty of Science and Technology, Omdurman Islamic University, Salha St., Khartoum 14415, Sudan; khalidbrema57@gmail.com; 8 Department of Software Engineering, Faculty of Computer Science, Al-Neelain University, El Gamhuriya Avenue, Khartoum 11121, Sudan

**Keywords:** *PAPPA*, pan-cancer, tumor microenvironment, cancer-associated fibroblasts, promoter methylation, prognostic biomarker

## Abstract

Pregnancy-associated plasma protein A (*PAPPA*) is a metalloproteinase that regulates insulin-like growth factor availability via cleavage of IGF-binding proteins, yet its role in cancer remains incompletely understood. Using integrated public datasets, we systematically examined *PAPPA* expression, prognostic relevance, cellular localization, and stromal associations across multiple tumor types. *PAPPA* was reduced in several cancers and primarily localized to stromal cells, whereas in cholangiocarcinoma and thyroid carcinoma it was elevated and also detected in malignant cells. High *PAPPA* expression was associated with poorer overall survival in bladder, cervical, lung squamous, mesothelioma, pancreatic, and gastric cancers, but exhibited a protective effect in lower-grade glioma. In tumors with adverse prognosis, *PAPPA* strongly correlated with cancer-associated fibroblast (CAF) infiltration and CAF marker genes; however, multivariable Cox analyses indicated that *PAPPA* generally retained an independent prognostic factor, whereas CAF infiltration was mostly not independently associated with overall survival. Interestingly, in LGG, despite negative *PAPPA*–CAF correlations, multivariable analysis showed that *PAPPA* remained protective while CAF infiltration was associated with worse survival. Pathway analyses linked *PAPPA*-associated genes to proteoglycans in cancer and PI3K–AKT and RAS signaling. Collectively, these findings establish *PAPPA* as an independent prognostic factor across most cancers, while its expression frequently coincides with high CAF infiltration in select tumor types, highlighting the need for further investigation.

## Simple Summary

Pregnancy-associated plasma protein A (*PAPPA*) is important for normal fetal development, but its exact role in cancer has been unclear. In this study, we analyzed *PAPPA* across many cancer types to understand how it relates to tumor progression and patient survival. We found that in many tumors, *PAPPA* is produced not by the cancer cells themselves but by surrounding connective or stromal cells, which can help shape a tumor-supportive environment. *PAPPA* is linked to worse outcomes in most cancers, while in others it is associated with better survival. Higher *PAPPA* levels in cancers such as pancreatic and lung cancer were generally associated with poorer prognosis, whereas in certain brain tumors they were linked to improved survival. These findings suggest that *PAPPA* may serve as a useful biomarker for assessing tumor aggressiveness and highlight the importance of the tumor microenvironment. Understanding how cancers interact with nearby healthy cells may help clinicians better predict patient risk and inform strategies that target this supportive environment.

## 1. Introduction

Originally identified as a circulating placental antigen abundant in pregnancy, pregnancy-associated plasma protein A (*PAPPA*) is now recognized as a zinc-binding metalloproteinase with critical regulatory functions. *PAPPA* is a critical activator of the insulin-like growth factor (IGF) pathway, primarily through its ability to cleave IGF-binding protein-4 (IGFBP-4), thereby releasing IGFs to activate downstream signaling cascades such as the PI3K–AKT pathway [1,2,3,4]. This activation has broad implications, including roles in cell proliferation, survival, and inflammation. *PAPPA* is widely expressed in normal tissues, with the highest expression levels observed in the kidney and bone [5].

Despite its established role in normal physiology, its function in cancer remains controversial, with evidence supporting both oncogenic and tumor-suppressive properties. Numerous studies have demonstrated that *PAPPA* is overexpressed in several cancers, such as breast, ovarian, and lung cancers and Ewing sarcoma, where it acts as an oncogene, promoting tumorigenesis, invasion, and resistance to therapies [6,7,8,9]. Conversely, other studies have suggested that *PAPPA* functions as a tumor suppressor in specific cancer types. For example, it is epigenetically silenced in precursor lesions and invasive breast cancers, with its downregulation associated with enhanced invasiveness and chromosomal instability [10]. Similarly, in renal cell carcinoma, low *PAPPA* expression is linked to poor prognosis, whereas *PAPPA* overexpression has been shown to inhibit cell proliferation, migration, and invasion [11]. These findings suggest a context-specific role for *PAPPA*, potentially influenced by cancer type, tumor stage, and the surrounding microenvironment.

The conflicting evidence regarding *PAPPA*’s role in cancer highlights the need for a comprehensive understanding of its expression patterns and effect on prognosis across various cancer types. To address this complexity, we conducted a pan-cancer analysis to systematically examine *PAPPA* expression patterns, clinical relevance, prognostic value, immune infiltration, genetic alterations, and molecular interactions using The Cancer Genome Atlas (TCGA) and additional bioinformatic methods. Our study aimed to provide an integrated characterization of *PAPPA* and gauge its capacity to serve as a predictive clinical marker in diverse cancer types.

## 2. Materials and Methods

Details regarding bioinformatic resources, database specifications and modules utilized (Supplementary Table S1), processing of microarray validation datasets, immunohistochemical staining evaluation, survival analysis and statistical parameters, functional enrichment and network construction are provided in Supplementary Information.

*Integrated expression profiling and clinical association.* To systematically evaluate *PAPPA* dysregulation across diverse malignancies, we employed a multi-database mining strategy. Initial tumor-normal differential expression screenings were conducted using the TIMER2.0 (https://compbio.cn/timer2/, accessed on 2 February 2025) [12] and GEPIA2 (http://gepia2.cancer-pku.cn/, accessed on 2 February 2025) [13] platforms, followed by detailed stratification based on clinical parameters and promoter methylation status via UALCAN (https://ualcan.path.uab.edu/, accessed on 2 February 2025) [14,15]. All tumor expression analyses were based on TCGA project cohorts and reflect primary tumors at the site of origin; metastatic lesions were not analyzed as a separate category. The criteria for further analysis required consistent expression patterns across multiple independent datasets with statistically significant contrasts, whereby only cancer types that were found to be dysregulated in a similar fashion in at least two databases were further analyzed.

*Single-cell characterization and tissue validation.* To map transcriptomic signatures to specific cellular lineages within the tumor microenvironment (TME), single-cell transcriptomic profiles were retrieved and analyzed via the TISCH2 repository (http://tisch.comp-genomics.org/, accessed on 8 February 2025) [16]. These profiles were contextualized against baseline physiological expression in non-cancerous tissues using the GTEx project (https://gtexportal.org/home/, accessed on 8 February 2025) [17]. Furthermore, transcriptional findings were corroborated at the proteomic level by evaluating immunohistochemical (IHC) staining patterns from the Human Protein Atlas [18,19]. Tissue microarrays were assessed for staining intensity and subcellular localization to confirm tumor-specific protein expression.

*Independent validation and statistical analysis.* Validation of expression patterns was performed using external microarray cohorts retrieved from the Gene Expression Omnibus [20,21]. Data processing involved rigorous normalization and differential expression testing using the linear models for microarray data (*limma*) framework [22], applying the Benjamini–Hochberg correction procedure to maintain a controlled false discovery rate. Comprehensive details regarding data processing and statistical parameters are provided in Supplementary Information.

*Prognostic modeling and survival analysis.* The clinical relevance of *PAPPA* was assessed by modeling its association with patient survival outcomes. Kaplan–Meier survival estimates and hazard ratios were generated using the Kaplan–Meier Plotter (https://kmplot.com/analysis, accessed on 8 February 2025) [23,24] and validated using TISCH2 and GEPIA2. Samples were dichotomized into groups with elevated versus diminished transcript levels using the median gene expression value as the cutoff to determine the prognostic impact on overall and disease-free survival, unless the database algorithm specified an optimal cutoff. Samples from TISCH2 are derived from TCGA datasets, rather than from single-cell sequencing samples themselves [16].

*Immune landscape and genomic alterations.* To characterize the immunological landscape, we quantified the correlation between gene expression and the abundance of tumor-infiltrating immune cells (TIICs) using the TIMER algorithm implemented in TIMER, where gene–cell correlations were assessed using purity-adjusted Spearman correlation coefficients, as provided by the Gene module [25,26]. In parallel, genomic stability was investigated by analyzing somatic mutations and copy number variations (CNVs) via the cBioPortal for Cancer Genomics [27,28,29], allowing for the assessment of genetic alterations associated with *PAPPA* dysregulation.

*Functional enrichment and network analysis.* Potential biological mechanisms were elucidated by constructing protein–protein interaction networks utilizing the STRING (https://string-db.org/, accessed on 7 February 2025) [30,31] and GeneMANIA (http://genemania.org/, accessed on 7 February 2025) [32] databases. A consensus gene set derived from these interactions was subjected to Gene Ontology (GO) and KEGG pathway enrichment analyses using Enrichr (https://maayanlab.cloud/Enrichr/, accessed on 7 February 2025) [33,34,35] to identify key signaling cascades and biological processes. Finally, the role of stromal components was investigated by analyzing the infiltration of cancer-associated fibroblasts (CAFs), and their correlation with established marker genes using the TIMER algorithm in TIMER2.0 (https://compbio.cn/timer2/, accessed on 15 February 2025).

*Cox risk regression analysis.* Cox multivariable regression analyses were performed using the TIMER2.0 platform (accessed on 23 January 2026) to evaluate the independent effects of PAPPA expression and tumor purity on overall survival (OS) using the model *Surv(OS, EVENT) ~ Purity + PAPPA*. A second model incorporating cancer-associated fibroblast (CAF) infiltration (*Surv(OS, EVENT) ~ Infiltrate + Purity + PAPPA*) was additionally examined, and the resulting estimates were visualized as forest plots in R (version 4.3.1) using the “forestplot” package.

## 3. Results

### 3.1. PAPPA Gene Expression in Pan-Cancer Analysis

The differences in *PAPPA* gene expression levels between tumor and normal tissues were analyzed using data from three independent databases (Supplementary Table S2). Tumor Immune Estimation Resource 2 (TIMER2.0), Gene Expression Profiling Interactive Analysis 2 (GEPIA2), and University of Alabama at Birmingham Cancer Data Analysis Portal (UALCAN). This multi-database approach ensured the robustness of findings, with significant differential expression determined based on consistent results observed in at least two of the three databases.

**Figure 1:**
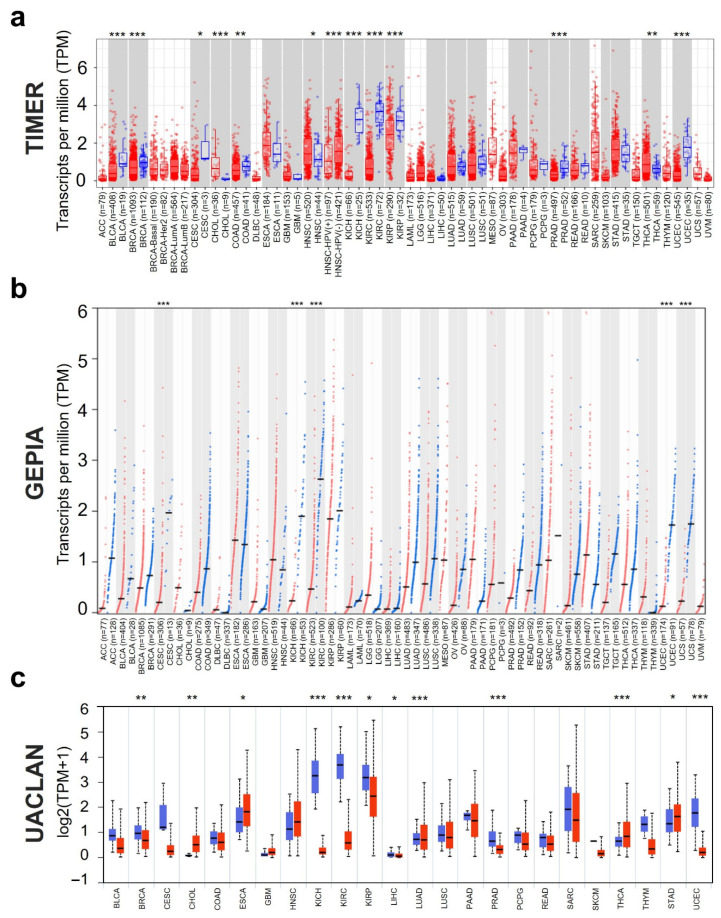
*PAPPA* expression across different cancer types: (**a**–**c**) *PAPPA* mRNA expression in tumor (red) and normal (blue) tissues based on the TIMER2.0 (**a**), GEPIA2 (**b**), and UALCAN (**c**) databases. Differential expression significance was computed by differential analysis (edgeR) on RNA-Seq Raw counts in TIMER2.0, the LIMMA method with *p*-value-based filtering in GEPIA (|log2FC| ≥ 1.5, *p*-value ≤ 0.05; TCGA normal and GTEx reference), and Welch’s *t*-test in UALCAN. Statistical significance is indicated by asterisks (*: *p* < 0.05; **: *p* < 0.01; ***: *p* < 0.001).

The TIMER2.0 analysis revealed significant differences in *PAPPA* expression across 12 tumor types. Notably, *PAPPA* expression was significantly upregulated in cholangiocarcinoma (CHOL) (*p* < 0.001), head and neck squamous cell carcinoma (HNSC) (*p* < 0.05), and thyroid carcinoma (THCA) (*p* < 0.01) but downregulated in the remaining nine cancers, namely bladder urothelial carcinoma (BLCA) (*p* < 0.001), breast invasive carcinoma (BRCA) (*p* < 0.001), cervical squamous cell carcinoma and endocervical adenocarcinoma (CESC), colon adenocarcinoma (*p* < 0.01), kidney chromophobe (KICH) (*p* < 0.001), kidney renal clear cell carcinoma (KIRC) (*p* < 0.001), kidney renal papillary cell carcinoma (KIRP) (*p* < 0.001), prostate adenocarcinoma (PRAD) (*p* < 0.001), and uterine corpus endometrial carcinoma (UCEC) (*p* < 0.001) (Figure 1a).

To validate these findings, we analyzed *PAPPA* expression using the GEPIA2 platform, which identified five cancers, namely CESC, KICH, KIRC, UCEC, and uterine carcinosarcoma (UCS), with significantly different *PAPPA* expression between tumor tissues and adjacent normal tissues. Specifically, all five cancers exhibited significantly downregulated *PAPPA* expression (*p* < 0.001) in tumor tissues (Figure 1b). Notably, all GEPIA2-identified cancers except UCS were also detected by TIMER2, demonstrating strong inter-database concordance.

Further confirmation was obtained using the UALCAN platform. This platform validated *PAPPA* upregulation in CHOL and THCA (*p* < 0.05) and downregulation in BRCA, KICH, KIRC, KIRP, PRAD, and UCEC (all *p* < 0.05). Interestingly, UALCAN uniquely identified significantly reduced *PAPPA* expression in stomach adenocarcinoma (STAD) (*p* < 0.001), which was not detected in the other two databases (Figure 1c).

**Figure 2:**
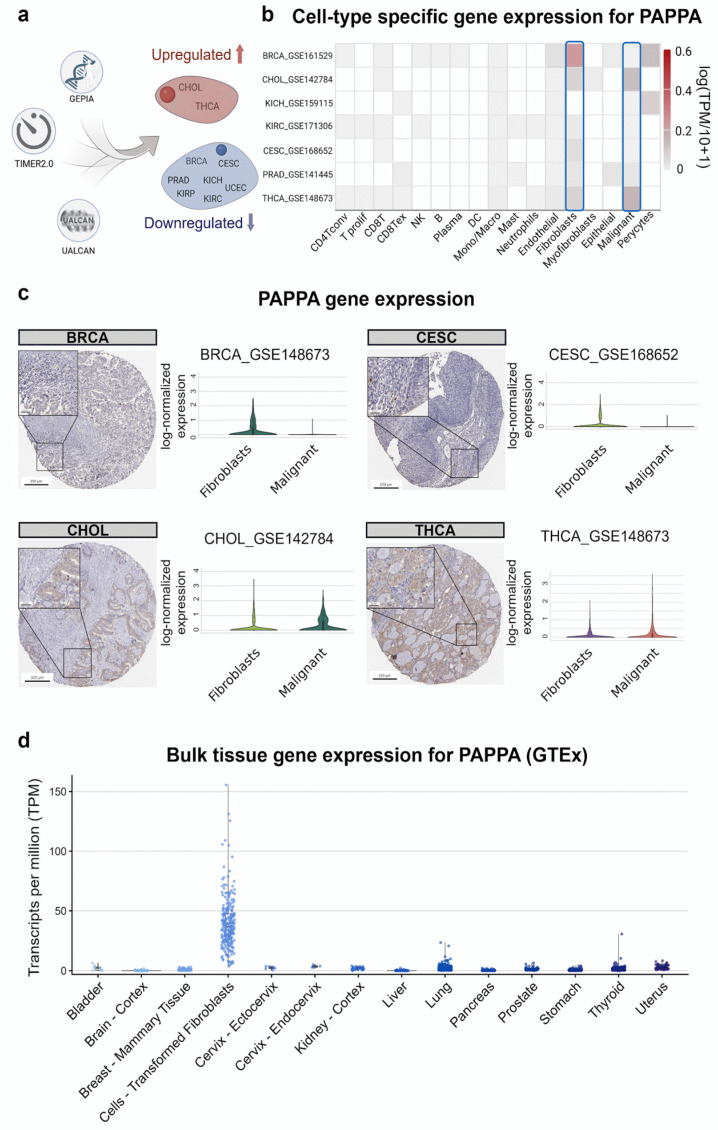
*PAPPA* single-cell expression and tissue analyses. (**a**) Workflow summarizing the integrative analysis using three databases (TIMER2, GEPIA2, and UALCAN). (**b**) Heatmap of scRNA-seq-determined *PAPPA* expression across cancer types from the TISCH2 database. The blue box highlights malignant cells and fibroblasts, indicating the cell populations analyzed for comparison. (**c**) Representative IHC images (left) show PAPPA protein levels in BRCA, CESC, CHOL, and THCA tumor sections. Analysis of BRCA and CESC samples revealed predominant *PAPPA* immunoreactivity in fibroblasts, with no immunoreactivity in tumor cells. In contrast, CHOL and THCA specimens exhibited *PAPPA* expression exclusively in tumor cells, with no detectable staining in the stromal compartment. Corresponding violin plots (right) display single-cell *PAPPA* expression using TISCH2 datasets. Violin plots show Seurat log-normalized expression values (log1p of Unique Molecular Identifier (UMI) counts normalized to 10,000 per cell) across fibroblasts and malignant cells, illustrating the cell-type distribution of *PAPPA* expression within each tumor type. (**d**) Violin plot of *PAPPA* expression across bulk normal tissues from the GTEx portal, confirming fibroblasts as the primary source of expression.

For further analysis, we prioritized cancers that showed consistent changes in *PAPPA* expression in at least two databases, which corresponded to TCGA-defined primary tumor entities. These included two cancers with upregulated *PAPPA* expression (CHOL and THCA) and seven with downregulated *PAPPA* expression (BRCA, CESC, KICH, KIRC, KIRP, PRAD, and UCEC) (Figure 2a).

### 3.2. Single-Cell Expression Analysis Highlights a Stromal Origin for PAPPA

Given that *PAPPA* expression was largely downregulated in bulk tumor tissues, we investigated its cellular source using single-cell RNA sequencing (scRNA-seq) to determine the tumor microenvironment compartments responsible for its expression. The Tumor Immune Single-cell Hub 2 (TISCH2) database analysis revealed that *PAPPA* expression was predominantly localized to fibroblasts across multiple cancer types, with minimal or undetectable expression in malignant cells, except in CHOL and THCA (Figure 2b). A comprehensive view of *PAPPA* expression across all cell types from TISCH2 is provided in Supplementary Figure S1.

To determine the cellular source of *PAPPA* in cancer tissues, we examined immunohistochemistry (IHC) images from the Human Protein Atlas. The observed staining patterns were consistent with our single-cell expression analysis across multiple cancer types (Figure 2c). In BRCA and CESC, *PAPPA* staining was predominantly detected in stromal, non-malignant cells, supporting the low *PAPPA* expression levels found in tumor cells from these cancers. In contrast, in CHOL and THCA, *PAPPA* staining was primarily localized to cancer cells. To further validate the IHC results, we performed quantitative analysis of single-cell gene expression in malignant and fibroblast cells using TISCH2. The results are shown as violin plots displayed in Figure 2c. In BRCA and CESC, *PAPPA* expression was higher in fibroblasts (42.3% and 15.1%, respectively) than in malignant cells, which remained below the detection threshold. Conversely, CHOL and THCA exhibited higher expression in malignant cells (69.9% and 67.9%, respectively) and lower expression in fibroblasts (47.7% and no significant signal, respectively).

To further assess stromal contribution at the bulk-tumor level, we performed tumor purity analysis using TIMER2.0. This revealed a predominantly significant negative correlation between *PAPPA* expression and tumor purity in dysregulated cancers, including BRCA (r = −0.29, *p* < 0.001), CESC (r = −0.19, *p* = 0.001), KICH (r = −0.28, *p* < 0.05), PRAD (r = −0.41, *p* < 0.001), and UCEC (r = −0.16, *p* < 0.01). THCA also showed a negative association, although it represented the weakest correlation among the statistically significant cancers (r = −0.09, *p* < 0.05). In contrast, CHOL, KIRC, and KIRP exhibited weaker, non-significant correlations.

To confirm that fibroblasts also represent the principal non-malignant source of *PAPPA* under physiological conditions, we interrogated the Genotype-Tissue Expression (GTEx) database, which similarly demonstrated predominant *PAPPA* expression in fibroblast populations (Figure 2d). Collectively, these findings support stromal enrichment and suggest that bulk tumor PAPPA levels largely reflect fibroblast abundance rather than tumor cell–intrinsic transcriptional regulation.

### 3.3. Clinical Features of PAPPA Expression

**Figure 3:**
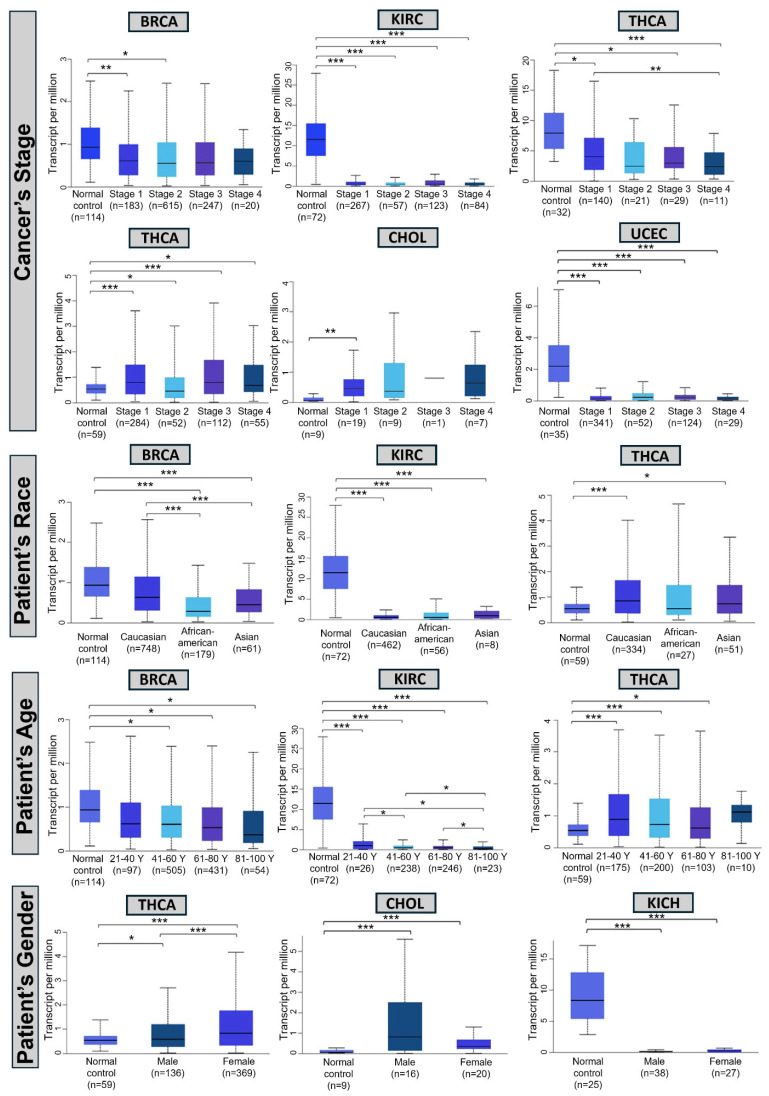
Clinical parameters of *PAPPA* expression across cancer types analyzed using the UALCAN database (Welch’s *t*-test). Box plots depict *PAPPA* expression levels (transcripts per million, TPM) in normal versus tumor tissues, stratified by cancer stage, race, age, and sex. Sample sizes for each group (n) are indicated. Statistical significance is denoted by asterisks (*: *p* < 0.05; **: *p* < 0.01; ***: *p* < 0.001).

To contextualize *PAPPA* within a clinical framework, we analyzed its relationship to various clinical parameters using the UALCAN database. This analysis included comparisons by tumor stage, patient race, age, and sex (Figure 3). At advanced disease stages, *PAPPA* expression differed significantly between tumor and normal tissues in BRCA, CHOL, KIRC, KIRP, THCA, and UCSC. Among these, KIRP was the only cancer type exhibiting significant intra-stage variation, with *PAPPA* expression markedly reduced in stage IV malignancies relative to stage I lesions (*p* < 0.01).

In racial comparisons, distinct *PAPPA* expression patterns were observed in BRCA, KIRC, and THCA across different races. Notably, marked disparities were observed between malignant and adjacent normal samples in KIRC and THCA across racial groups. In contrast, BRCA uniquely displayed variation between races, with *PAPPA* expression in Caucasian patients differing significantly from those in both African American and Asian patients (both with *p* < 0.001).

Age-related variations in *PAPPA* expression were also observed in BRCA, KIRC, and THCA when compared with normal tissues. Among these, KIRC uniquely exhibited significant differences among the age groups. Specifically, *PAPPA* expression was significantly lower in younger individuals (21–40 years) than in older groups (41–60 years and 61–80 years; *p* < 0.05). Furthermore, a progressive decline in *PAPPA* expression was observed with advancing age, as intermediate age groups (41–60 years and 61–80 years) exhibited decreased expression compared with the oldest cohort (81–100 years; *p* < 0.05).

Finally, sex-associated differences were observed in CHOL, KICH, and THCA, with all three cancers showing significant divergence in transcript abundance between cancerous and non-neoplastic tissues. THCA was the only cancer additionally displaying significant expression differences between men and women (*p* < 0.001). Additional clinical parameters are provided in Supplementary Figure S2.

### 3.4. Methylation Status and Its Correlation with PAPPA Expression

To investigate potential epigenetic regulation of *PAPPA* among dysregulated cancers, we analyzed its promoter methylation status across cancer types using UALCAN (tumor–normal group comparisons) (Supplementary Figure S3). The *PAPPA* promoter exhibited significantly lower methylation levels in CHOL (*p* < 0.05) and THCA (*p* < 0.001) than in normal tissues, which aligned with the upregulated *PAPPA* gene expression observed in these cancers. In contrast, in KIRC (*p* < 0.05) and KIRP (*p* < 0.001), despite lower promoter methylation, *PAPPA* expression was paradoxically downregulated. For the remaining cancers analyzed (BRCA, CESC, KICH, PRAD, and UCS), no significant differences in promoter methylation were observed. To further evaluate whether promoter methylation was associated with transcriptional output, sample-level expression–methylation correlations were subsequently examined using TCGA PanCancer Atlas datasets in cBioPortal for cancers showing significant group-level differences (Supplementary Figure S4). A significant inverse association was observed only in THCA (Spearman r = −0.32, *p* < 0.001), whereas CHOL, KIRC, and KIRP showed non-significant correlations.

### 3.5. PAPPA Gene Expression from Clinical GEO Datasets

**Figure 4:**
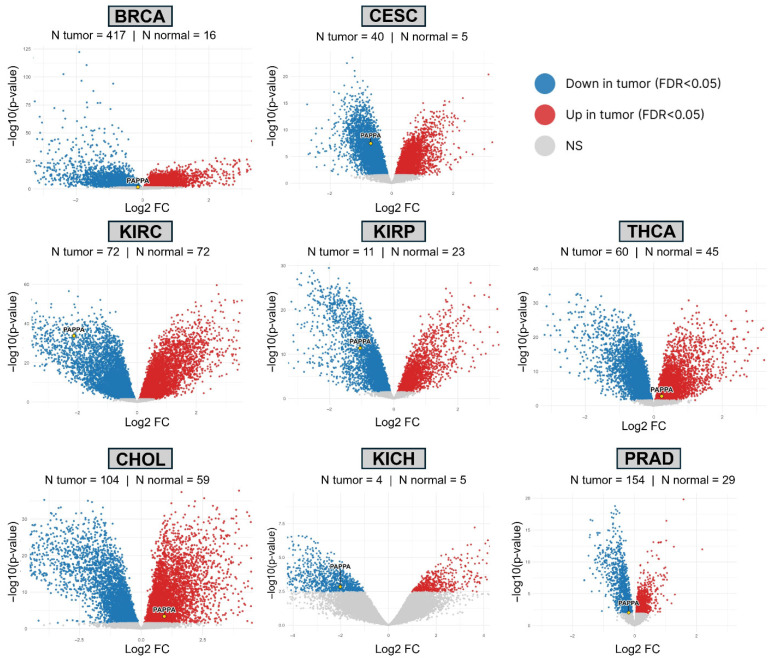
Volcano plots summarizing differential expression between tumor and normal tissues across eight GEO cohorts. Each point is a gene; the *x*-axis shows log_2_ fold change (Tumor − Normal), and the *y*-axis shows −log_10_ (*p* value). Red points denote significant upregulation in tumors (FDR < 0.05), blue points indicate significant downregulation, and gray points indicate no significant difference. *PAPPA* is labeled and highlighted. Sample sizes for tumor and normal groups are indicated in each panel.

To assess the reproducibility of *PAPPA* deregulation, we analyzed eight Gene Expression Omnibus (GEO) datasets covering BRCA (GSE54002), CESC (GSE7410), KICH (GSE11151), KIRC (GSE53757), KIRP (GSE15641), PRAD (GSE21034), CHOL (GSE26566), and THCA (GSE33630). Each cohort included both primary tumor samples, metastatic samples and non-malignant samples profiled on microarray platforms. All identified tumor samples, including metastatic lesions, were included in the differential expression analysis. Genome-wide volcano plots were generated for every dataset, with *PAPPA* labeled to show its position relative to all other genes. Tumor and normal sample counts for each cohort are displayed in the panels (Figure 4).

Across these datasets, *PAPPA* expression showed a uniform direction of change for each cancer type. Compared with normal tissue, BRCA, CESC, KICH, KIRC, KIRP, and PRAD demonstrated lower *PAPPA* expression in tumors, whereas CHOL and THCA showed higher *PAPPA* expression. Kidney cohorts displayed the largest fold changes, supported by tumor–normal sampling. Cohorts with fewer normal samples, such as BRCA and PRAD, had smaller effect sizes but retained statistical significance.

Differences in effect magnitude between datasets reflect underlying tissue composition, sample size, and platform characteristics. Studies with matched or near-matched tumor–normal sampling, particularly the kidney cohorts, produced clear contrasts. Cancers with limited availability of normal controls showed narrower fold-change distributions, but the direction of *PAPPA* expression change remained stable.

These cross-cohort comparisons confirm that *PAPPA* deregulation is consistent across independent patient datasets. Reduced expression in several epithelial and renal cancers, along with increased expression in CHOL and THCA, supports tumor-type-specific regulation of *PAPPA*.

### 3.6. Overall Survival Analysis of PAPPA Expression

To evaluate the effect of *PAPPA* expression on patient prognosis, overall survival (OS) analyses were performed by stratifying patients into high- and low-expression groups using three independent databases: TISCH2, GEPIA2, and Kaplan–Meier Plotter.

**Figure 5:**
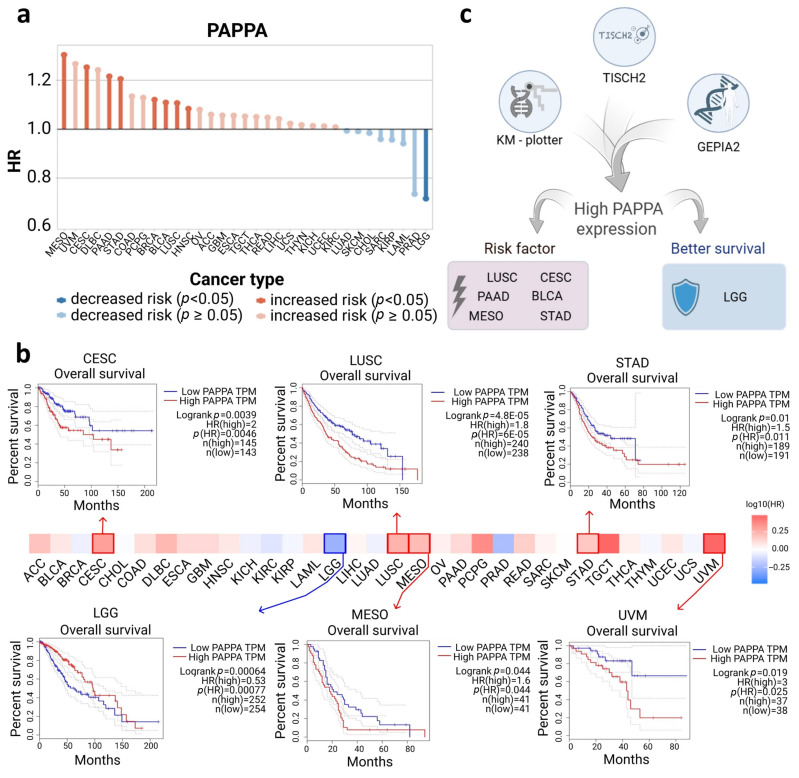
Association between *PAPPA* expression and OS across cancer types. (**a**) Integrated HR analysis across different cancer types. Solid red bars show that high *PAPPA* expression increases the risk of death, whereas solid blue bars show that high *PAPPA* expression decreases the risk of death. (**b**) Kaplan–Meier survival curves from the GEPIA2 database utilizing KM analysis and log rank test, with high expression shown in red and low expression shown in blue. (**c**) Workflow summarizing the integrated OS analysis using GEPIA, Kaplan–Meier Plotter, and TISCH2, identifying cancers in which high *PAPPA* expression is consistently associated with poor (red) or improved (blue) prognosis.

TISCH2 identified nine significant cancers (Figure 5a). High *PAPPA* expression functions as a risk factor in eight of them: mesothelioma (MESO), CESC, PAAD, and STAD (*p* < 0.05, hazard ratio [HR] ≥ 1.2), as well as BRCA, BLCA, LUSC, and HNSC, as cancers in which elevated *PAPPA* was similarly associated with poor prognosis (*p* < 0.05, 1.2 < HR < 1.5). In contrast, lower-grade glioma (LGG) remained the only cancer where high *PAPPA* expression correlated with better survival (*p* < 0.05, 0.6 < HR < 0.8).

GEPIA2 identified five major cancers (Figure 5b) in which elevated *PAPPA* levels predicted shorter overall survival: CESC (*p* < 0.01, HR = 2.0), lung squamous cell carcinoma (LUSC; *p* < 0.001, HR = 1.8), (MESO; *p* < 0.05, HR = 1.6), STAD (*p* < 0.05, HR = 1.5), and uveal melanoma (UVM; *p* < 0.05, HR = 3.0). Consistent with TISCH2, LGG (*p* < 0.001, HR = 0.53) showed that high *PAPPA* expression correlated with better OS.

Kaplan–Meier Plotter identified five cancers (Supplementary Figure S5), all showing high *PAPPA* expression as the risk factor: BLCA (*p* < 0.05, HR = 1.11), CESC (*p* < 0.001, HR = 1.24), LUSC (*p* < 0.05, HR = 1.10), PAAD (*p* < 0.05, HR = 1.17), and STAD (*p* < 0.01, HR = 1.21).

Integrative survival analysis revealed seven cancers identified by at least two databases (Figure 5c). Among these, six cancers (BLCA, CESC, LUSC, MESO, PAAD, and STAD) consistently showed poor prognosis associated with high *PAPPA* expression. In contrast, in LGG, high *PAPPA* expression predicted improved OS, highlighting a distinct, tumor-specific role of PAPPA.

### 3.7. Correlation of PAPPA with Immune Cell Infiltration

Immune infiltration analysis revealed distinct cancer-specific associations between *PAPPA* expression and the tumor immune landscape. To explore whether *PAPPA* levels were linked to immune composition, TIMER analysis was performed (Supplementary Table S3 and Figure S6).

In BLCA, PAPPA displayed an inverse relationship with tumor purity (r = −0.40, *p* < 0.001) and a positive correlation with dendritic cells (r = 0.38, *p* < 0.001). In LUSC, PAPPA expression was likewise negatively correlated with purity (r = −0.31, *p* < 0.001) and positively associated with neutrophils (r = 0.30, *p* < 0.001). PAAD exhibited the most pronounced immune-enriched profile, with strong positive correlations with macrophages (r = 0.56, *p* < 0.001) and CD8^+^ T cells (r = 0.51, *p* < 0.001).

In contrast, LGG demonstrated an opposite pattern, with *PAPPA* positively correlated with tumor purity (r = 0.13, *p* = 0.004) but negatively associated with dendritic cells (r = −0.21, *p* < 0.001), indicating a distinct microenvironmental context. Other tumor types showed weaker or non-significant associations.

To determine whether these microenvironmental associations translated into independent survival effects, multivariable Cox regression analyses incorporating *PAPPA* expression and tumor purity were performed. The results largely mirrored the univariate OS trends, with *PAPPA* remaining a significant risk factor in BLCA (HR = 1.25, *p* < 0.05), CESC (HR = 1.83, *p* < 0.001), LUSC (HR = 1.20, *p* = 0.013), MESO (HR = 1.54, *p* < 0.001), and STAD (HR = 1.21, *p* < 0.05). Notably, PAAD, which previously exhibited the strongest immune and stromal infiltration pattern, lost statistical significance after purity adjustment. In contrast, LGG retained a significant protective association (HR = 0.55, *p* < 0.05), suggesting a biologically distinct, tumor cell-linked role of PAPPA in this context.

### 3.8. Genetic Alteration Analysis

**Figure 6:**
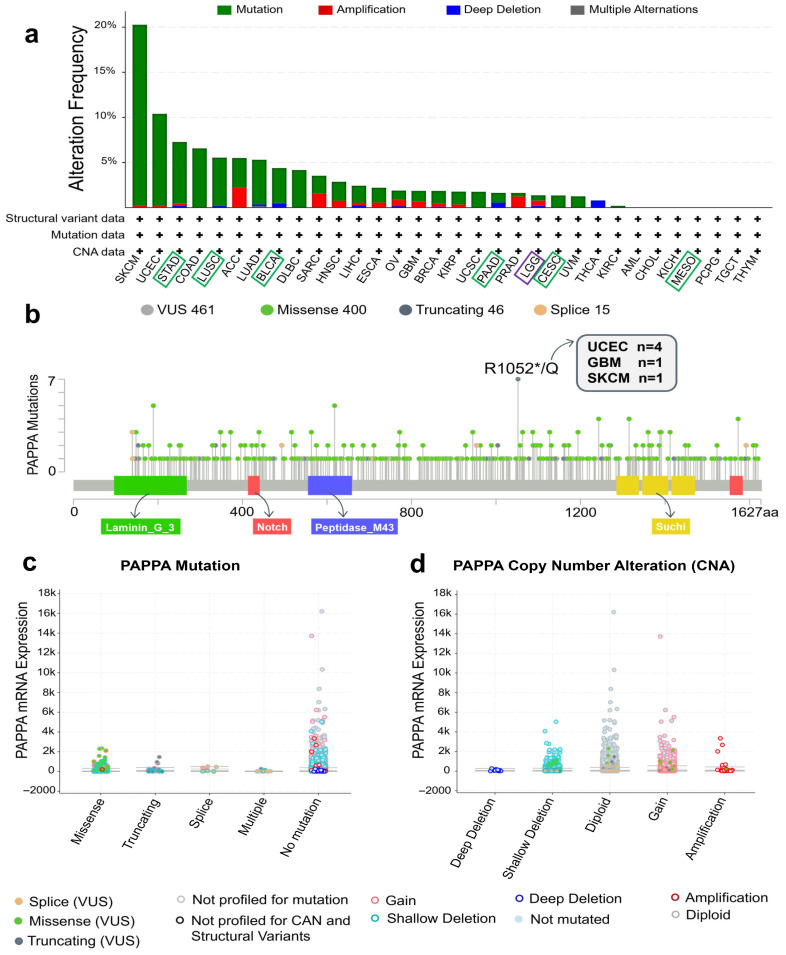
Genetic alterations of *PAPPA* in pan-cancer. (**a**) Summary of genetic alterations of *PAPPA* in TCGA Pan-Cancer Atlas studies. Green boxes indicate cancers in which high *PAPPA* expression is associated with poor prognosis, while the purple box indicates cancer in which high expression predicts better outcomes. (**b**) Mutation types, numbers, and sites of *PAPPA* across protein domains. (**c**) Correlation between the mutation types of *PAPPA* and its expression in cancers. (**d**) Correlation between the putative CNA of *PAPPA* and its expression in cancers.

We used cBioPortal to investigate possible genetic mechanisms of *PAPPA* dysregulation in cancer. Across 10,967 samples covering 32 TCGA tumor types, we assessed *PAPPA* for alteration frequency, specific mutation types, and copy number alterations (CNAs). Genetic alterations in the *PAPPA* gene have been identified across 25 different tumor types. Among these alterations, mutations were the most frequent, followed by amplifications. Notably, melanoma, despite not showing any dysregulation in *PAPPA* expression compared to normal tissue, had the highest mutation frequency (20%). Among the seven identified prognostic cancers, the mutation frequency was relatively low: STAD (7%), LUSC (6%), and the remaining cancers were below 5%. Interestingly, LGG, which is the only cancer where high *PAPPA* expression is associated with an improved prognosis, exhibited the highest amplification frequency among these seven cancers (0.6%) (Figure 6a). A total of 461 *PAPPA* mutation sites were identified across TCGA samples. For instance, a truncating mutation (R1052*/Q) was detected in UCEC, GBM, and SKCM (Figure 6b). Missense mutations showed the strongest association with changes in *PAPPA* mRNA levels, followed by truncating mutations (Figure 6c).

The CNA analysis revealed that shallow deletions were the most common alterations affecting *PAPPA*, whereas deep deletions were less frequent and showed only a minimal impact on *PAPPA* mRNA expression (Figure 6d). Thereafter, we evaluated the effect of genetic alterations of *PAPPA* on the clinical outcomes of patients. No significant differences in OS or disease-free survival were observed between the altered and non-altered groups (Supplementary Figure S7).

### 3.9. PAPPA Enrichment Analysis

To gain a comprehensive understanding of the biological functions of *PAPPA*, we analyzed its interaction networks using the STRING and GeneMANIA databases.

**Figure 7:**
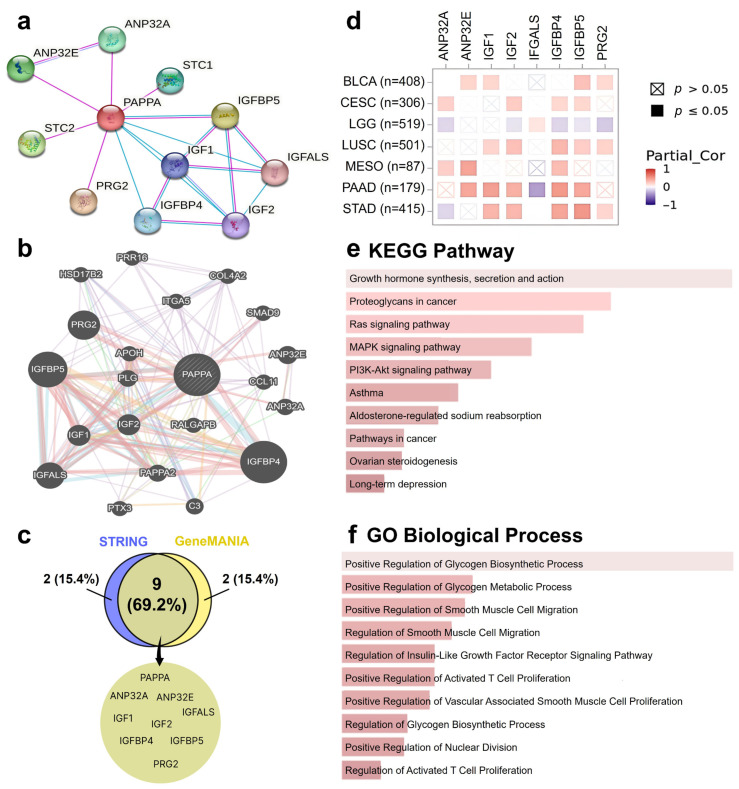
Analysis of *PAPPA*-associated genes and enriched functional pathways. (**a**) Protein–protein interaction network generated using STRING. Blue lines indicate known interactions derived from curated databases, while pink lines represent experimentally determined interactions. (**b**) Gene interaction network generated using GeneMANIA. (**c**) Venn diagram showing overlapping genes identified by both STRING and GeneMANIA analyses. (**d**) Heatmap illustrates the correlation between *PAPPA* expression and eight related genes across selected cancer types. Red squares indicate a positive correlation and blue squares indicate a negative correlation, with darker shades representing stronger correlation values. Significant correlations (*p* ≤ 0.05) are marked with filled squares, whereas non-significant correlations are denoted by crossed boxes. (**e**) KEGG pathway enrichment analysis of *PAPPA*-associated genes. (**f**) GO enrichment analysis categorizing *PAPPA*-related genes based on biological processes.

First, we constructed a protein–protein interaction network in STRING, identifying the top 10 proteins most closely associated with *PAPPA* (Figure 7a). A similar gene interaction network was generated in GeneMANIA (Figure 7b). To highlight overlapping partners across both platforms, we compared the two networks using a Venn diagram, which revealed nine common genes, including *PAPPA* itself (Figure 7c).

Next, we evaluated the correlation between *PAPPA* and these eight genes across multiple cancers using the TIMER2.0 database with tumor purity adjustment. The heatmap (Figure 7d) illustrated these correlations. Among all genes investigated, *IGFBP4*, *IGFBP5*, *IGF1*, were the most consistently prevalent, showing positive correlations across multiple cancers with the strongest correlations observed in STAD (*IGFBP5*: r = 0.50, *p* < 0.001; *IGFBP4*: r = 0.42, *p* < 0.001; *IGF1*: r = 0.34, *p* < 0.001), followed by PAAD (*IGFBP4*: r = 0.48, *p* < 0.001; *IGF1*: r = 0.45, *p* < 0.001; *IGFBP5*: r = 0.41, *p* < 0.001). Although *IGF1* was positively correlated with PAPPA, its correlation coefficients were consistently weaker than those observed for *IGFBP4* and/or *IGFBP5* within the same tumor types. Interestingly, in LGG, *PAPPA* expression showed predominantly negative correlations with six genes, including *IGFBP5* (r = −0.09, *p* < 0.05) and *IGFBP4* (r = −0.15, *p* < 0.001), while no significant correlation was observed with IGF1.

We next explored the molecular functions of *PAPPA* within the tumor context by utilizing Kyoto Encyclopedia of Genes and Genomes (KEGG) pathway enrichment analysis, which revealed strong associations of *PAPPA* with cancer and immune-related pathways, including growth hormone synthesis and secretion, proteoglycans in cancer, and oncogenic cascades such as RAS, MAPK, and PI3K–AKT signaling (Figure 7e). Consistently, Gene Ontology (GO) enrichment analysis demonstrated *PAPPA’s* involvement in the IGF receptor signaling pathway, regulation of glycogen metabolism, and regulation of smooth muscle cell migration and proliferation (Figure 7f).

### 3.10. PAPPA and Cancer-Associated Fibroblasts

**Figure 8:**
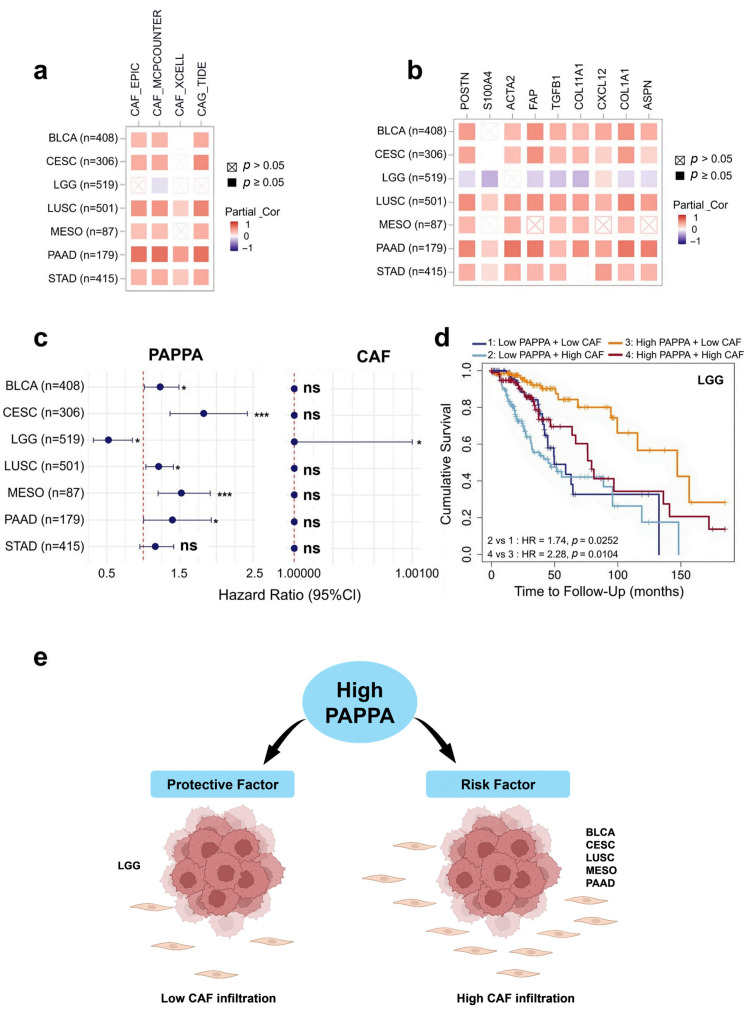
Correlation between *PAPPA* and CAF infiltration. (**a**) Correlation between *PAPPA* expression and CAF estimated using multiple *CAF* gene signatures. (**b**) Correlation between *PAPPA* expression and specific *CAF* gene markers using Spearman correlation. Across all panels, red squares indicate positive correlations and purple squares indicate negative correlations, with darker shades representing stronger correlation values. Significant correlations are denoted by black squares (*p* ≤ 0.05), whereas non-significant correlations are indicated by crossed boxes. (**c**) Forest plot of multivariable Cox regression analyses comparing the prognostic impact of *PAPPA* expression and cancer-associated fibroblast (CAF) infiltration. Hazard ratios (HRs) with 95% confidence intervals (CIs) for overall survival (OS) are shown for each cancer type derived from Cox proportional hazards models adjusted for tumor purity. Points represent HR estimates and horizontal lines indicate 95% CIs; the vertical dashed line denotes HR = 1 (no effect). Asterisks indicate statistical significance (* *p* < 0.05, *** *p* < 0.001; ns, not significant). (**d**) Kaplan–Meier (KM) survival analysis of LGG patients stratified into four groups based on high/low *PAPPA* expression and high/low CAF infiltration (MCP-COUNTER). Overall survival was compared using log-rank tests, and hazard ratios (HRs) were estimated using Cox proportional hazards models. (**e**) Scheme illustrating the proposed relationship between *PAPPA* expression, prognosis, and CAF infiltration in cancers where *PAPPA* is a prognostic marker.

To further investigate the role of *PAPPA* in the tumor microenvironment, we assessed its association with cancer-associated fibroblasts (CAFs), a major stromal component implicated in immune suppression and tumor progression. CAF infiltration was estimated using TIMER2.0, and correlations between *PAPPA* expression and CAF levels were calculated with tumor purity adjustment across cancers identified from the overall survival analysis (Figure 8a). Only cancers that showed significant correlations in at least two CAF estimation algorithms were considered. The results from MCP-COUNTER algorithms demonstrated that *PAPPA* expression was significantly positively correlated with CAF infiltration in BLCA (r = 0.36, *p* < 0.001), CESC (r = 0.41, *p* < 0.001), LUSC (r = 0.53, *p* < 0.001), MESO (r = 0.32, *p* < 0.01), PAAD (r = 0.70, *p* < 0.001), and STAD (r = 0.40, *p* < 0.001). In contrast, LGG displayed a weak but significant negative correlation (r = −0.10, *p* < 0.05) (Supplementary Table S4).

Next, we performed CAF marker analysis using TIMER2.0 to assess the correlation between *PAPPA* expression and various CAF markers (Figure 8b). After tumor purity adjustment, the results demonstrated that CAF markers, including *POSTN*, *ACTA2*, *TGFB1*, and *COL1A1*, were positively correlated with *PAPPA* expression across all cancers in which high *PAPPA* expression was associated with poor prognosis. Among these cancers, PAAD showed the strongest associations, with a strong positive correlation observed for *ACTA2* (r = 0.64, *p* < 0.001), *COL1A1* (r = 0.64, *p* < 0.001), *FAP* (r = 0.62, *p* < 0.001), and *COL11A1* (r = 0.51, *p* < 0.001). In contrast, LGG displayed negative correlations between *PAPPA* expression and 7 CAF markers, including *S100A4* (r = −0.26, *p* < 0.001), *COL11A1* (r = −0.24, *p* < 0.001), *TGFB1* (r = −0.20, *p* < 0.001), *FAP* (r = −0.14, *p* < 0.01).

To assess whether *PAPPA*-associated effects depended on CAF abundance, multivariable Cox proportional hazards regression was performed using TIMER2.0, incorporating *PAPPA* expression and CAF infiltration, and the results are presented as a forest plot (Figure 8c). Across most cancers, *PAPPA* remained significantly associated with overall survival, whereas CAF infiltration was not significant. In LGG, *PAPPA* remained protective (HR = 0.52, *p* < 0.01) while CAF infiltration was associated with poorer survival (HR ≈ 1.00, *p* < 0.001). Kaplan–Meier analysis further supported this pattern, showing that high *PAPPA* expression was associated with improved survival, particularly in tumors with low CAF infiltration (Figure 8d). The combined relationship between *PAPPA* expression, CAF abundance, and patient prognosis is summarized in the schematic model (Figure 8e).

## 4. Discussion

*PAPPA* is a key enzymatic regulator of IGF signaling, cleaving IGFBP-4 to amplify local growth factor availability within the tumor microenvironment. However, its role in cancer remains ambiguous, with evidence supporting both oncogenic and tumor-suppressive activities [2,15]. The present pan-cancer analysis evaluated *PAPPA* gene expression patterns, prognostic significance, and its association with cancer-associated fibroblast (CAF) infiltration across multiple cancer types, highlighting the microenvironmental context.

*PAPPA* expression was significantly dysregulated across nine cancer types, being downregulated in seven cancers (BRCA, CESC, KICH, KIRC, KIRP, PRAD, and UCEC) and upregulated in two (CHOL and THCA). Independent GEO datasets largely validated these expression patterns. Single-cell RNA-seq analysis demonstrated that *PAPPA* was predominantly expressed in stromal fibroblasts rather than malignant epithelial cells in most tumors. In contrast, in CHOL and THCA, *PAPPA* expression was detected in both stromal and tumor cells, a finding further supported by immunohistochemical staining demonstrating *PAPPA* positivity within malignant cells.

Consistent with this cellular distribution, tumor-purity analysis revealed predominantly significant inverse correlations between *PAPPA* expression and tumor purity across dysregulated cancers, with THCA showing a comparatively weaker but still significant association, whereas CHOL, KIRC, and KIRP exhibited weak or non-significant correlations, indicating that bulk *PAPPA* levels in most tumors largely reflect stromal/fibroblast abundance rather than tumor cell–intrinsic transcription. This spatial distribution suggests that *PAPPA* is primarily a paracrine factor that may participate in influencing tumor growth, invasion, and immune regulation [8].

Promoter methylation analysis based on tumor–normal bulk comparisons revealed *PAPPA* hypomethylation in CHOL, THCA, KIRC, and KIRP, suggesting a context-dependent epigenetic contribution to its regulation. This pattern was consistent with *PAPPA* upregulation in CHOL and THCA, whereas KIRC and KIRP displayed reduced *PAPPA* expression despite lower promoter methylation. However, when assessed at the individual tumor sample level, expression–methylation correlation was significant only in THCA, whereas CHOL, KIRC, and KIRP showed non-significant correlations. Together, these findings indicate that promoter methylation alone does not fully explain *PAPPA* transcriptional variability and that cellular composition or additional regulatory mechanisms may contribute [36].

Survival analyses indicated that high *PAPPA* expression was associated with worse outcomes in BLCA, CESC, LUSC, MESO, PAAD, and STAD, whereas favorable outcomes were observed in LGG. Notably, these trends remained largely stable after adjustment for tumor purity, suggesting that *PAPPA*’s prognostic relevance is not merely a by-product of stromal proportion but reflects a partially independent biological signal.

Genetic alteration analysis revealed a low mutation frequency of *PAPPA*, with more than 86% of detected variants being missense. This observation is consistent with previous reports indicating a limited phenotypic impact of *PAPPA* mutations [2]. Gene interaction analyses showed generally stronger correlations with IGFBP4 and IGFBP5 and IGF1 across multiple cancers, suggesting a closer transcriptional association within the IGF-binding protein axis. KEGG pathway enrichment further identified proteoglycans in cancer, PI3K–AKT, and RAS signaling pathways as recurrent *PAPPA*-associated pathways [37].

A particularly noteworthy finding was the strong and consistent association between *PAPPA* expression and both CAF infiltration and canonical CAF marker genes across multiple cancers. In tumors with adverse outcomes, *PAPPA* levels correlated positively with CAF abundance and marker expression. Multivariable Cox regression accounting for CAF infiltration and tumor purity showed that *PAPPA* remained an independent prognostic factor in most cancers, whereas CAF infiltration was mostly not significant, indicating that PAPPA’s effect on survival is not fully explained by CAF abundance.

These observations are consistent with accumulating experimental evidence indicating that *PAPPA* functions as an active microenvironmental regulator rather than merely a passive stromal marker. Multiple mechanistic studies converge on the concept that *PAPPA* does not simply reflect fibroblast presence but actively participates in stromal remodeling processes that facilitate tumor expansion and invasion. For example, in gastric cancer, proteolytically active *PAPPA* secreted by tumor cells and CAFs increases local IGF bioavailability and “educates” adjacent quiescent fibroblasts toward pro-tumor phenotypes, thereby establishing a feed-forward loop that promotes tumor growth, invasion, and metastatic dissemination [38].

Parallel findings in non-small cell lung cancer demonstrate that *PAPPA* secretion is required to drive in vivo xenograft growth, while in vitro proliferation is unaffected; only expression that elevates serum *PAPPA* levels increases tumor weight [8]. Comparable patterns have been reported in hepatocellular carcinoma, where *PAPPA* is predominantly expressed by hepatic stellate cell–derived myofibroblasts, while malignant hepatocytes exhibit minimal expression, indicating that its tumor-promoting activity is largely mediated through paracrine stromal mechanisms [39].

Consistent with the role of *PAPPA* as a risk factor, experimental studies in ovarian cancer have demonstrated that elevated *PAPPA* promotes tumor growth and platinum resistance, while *PAPPA* neutralization enhances cisplatin sensitivity and improves therapy response [40,41].

Interestingly, in LGG, which generally has a favorable prognosis, although CAF infiltration was low and negatively correlated with *PAPPA* expression, Cox analysis showed that even this limited CAF presence was associated with poorer survival. Despite this, *PAPPA* remained a significant protective factor, with high expression linked to better outcomes, particularly in tumors with low CAF abundance. These results are supported by a prior study, indicating that CAFs can influence outcomes even in fibroblast-poor LGG tumors [42].

Collectively, our results suggest a model (Figure 8e) in which *PAPPA* functions as a prognostic risk factor in several cancers, often marking a stromal “bad neighborhood” characterized by high CAF abundance, while in other tumor contexts it shows a protective association that appears less dependent on stromal influence.

## 5. Conclusions

This pan-cancer study demonstrates that *PAPPA* is an independent prognostic biomarker whose biological and clinical effects vary across tumor types. In BLCA, CESC, LUSC, MESO, PAAD, and STAD, elevated *PAPPA* expression is associated with adverse outcomes and often coincides with high CAF infiltration, although CAF abundance itself is not consistently an independent risk factor. In contrast, in fibroblast-poor tumors such as LGG, higher *PAPPA* expression is linked to more favorable outcomes, whereas CAF infiltration is considered an adverse prognostic factor. Future studies integrating spatial, single-cell, and functional approaches are needed to clarify the cellular sources and regulatory mechanisms underlying *PAPPA* activity across tumor types.

## 6. Limitations

This study encompasses analyses that rely on publicly available databases and datasets; therefore, our findings require experimental wet-laboratory validation to establish their biological and functional significance.

## Data Availability

All datasets analyzed in this study are publicly available. TCGA data were obtained from the Genomic Data Commons portal (https://portal.gdc.cancer.gov/, accessed on 2 February 2025). GTEx data were accessed through the GTEx Portal (https://gtexportal.org/, accessed on 8 February 2025). GEO datasets were downloaded from the NCBI GEO repository (https://www.ncbi.nlm.nih.gov/geo/, accessed on 2 February 2025). All scripts used for data processing and analysis are available from the corresponding author upon request.

## Supplementary Materials

The following supporting information can be downloaded at: https://www.mdpi.com/article/10.3390/biology15060460/s1, Figure S1: *PAPPA* single-cell expression analysis across cancer types from the TISCH2 database; Figure S2: Clinical parameters of *PAPPA* expression across cancer types analyzed using the UALCAN database; Figure S3: *PAPPA* promoter DNA methylation and corresponding gene expression analyzed in upregulated cancers (CHOL and THCA); Figure S4: Kaplan–Meier curves for Overall Survival (OS) based on *PAPPA* expression levels; Figure S5: *PAPPA* gene expression level correlation with the immune cells infiltration level in BLCA, CESC, LGG, LUSC, MESO, PAAD and STAD using the TIMER database; Figure S6: *PAPPA* gene expression level correlation with the immune cells infiltration level in different cancer types using the TIMER database; Figure S7: Genetic alterations in the *PAPPA* altered group versus unaltered group and correlation with overall survival (OS) and disease-free survival (DFS). Supplementary Table S1. Modules used in each database and the query parameters applied in each analysis, and the access periods; Supplementary Table S2. Cancer types and the expression profile compared to normal tissue in GEPIA2, TIMER2.0, and UALCAN; Supplementary Table S3: Correlation between PAPPA expression and immune cell infiltration across selected cancers using TIMER2.0; Supplementary Table S4: Overview of PAPPA tumor-specific characteristics across selected cancers.

## Abbreviations

CAF	Cancer-associated fibroblast(s)
cBioPortal	cBioPortal for Cancer Genomics
CI	Confidence interval
CNV	Copy number variation
GEO	Gene Expression Omnibus
GEPIA2	Gene Expression Profiling Interactive Analysis 2
GO	Gene Ontology
GTEx Portal	Genotype-Tissue Expression Portal
HR	Hazard ratio
IHC	Immunohistochemistry
IGF1	Insulin-like growth factor 1
IGF2	Insulin-like growth factor 2
IGFALS	Insulin-like growth factor binding protein, acid labile subunit
IGFBP4	Insulin-like growth factor binding protein 4
IGFBP5	Insulin-like growth factor binding protein 5
KEGG	Kyoto Encyclopedia of Genes and Genomes
MAPK	Mitogen-activated protein kinase
OS	Overall survival
PAPPA (PAPP-A)	Pregnancy-associated plasma protein A
PI3K–AKT	Phosphatidylinositol 3-kinase–AKT signaling pathway
RAS	Rat sarcoma (RAS) signaling pathway
scRNA-seq	Single-cell RNA sequencing
STRING	Search Tool for the Retrieval of Interacting Genes/Proteins
TCGA	The Cancer Genome Atlas
TGFB1	Transforming growth factor beta 1
TIICs	Tumor-infiltrating immune cells
TIMER2.0	Tumor Immune Estimation Resource 2.0
TISCH2	Tumor Immune Single-cell Hub 2
TME	Tumor microenvironment
UALCAN	The University of ALabama at Birmingham CANcer data analysis Portal
