# Supplementary material for: Pan-Cancer Analysis of PAPPA Gene Reveals Tumor-Specific Prognostic Effects
Source: Biology (Basel). 2026 Mar 12;15(6):460. doi: 10.3390/biology15060460 (PMC13024262; doi:10.3390/biology15060460)
Supplement: Supplementary file 1 [file biology-15-00460-s001.zip › biology-4097456-supplementary.pdf]

Pan-Cancer Analysis of *PAPPA* Reveals Tumor-Specific Prognostic Effects

Samah Mutasim Alfadul,[1,†] *Khalid Omama,*[2,†] *Alisa Y. Potapova,*[1] *Pavel A. Ivanov-Rostovtsev,*[1] *Maryam Fanian,*[1] *Reem Mubarak,*[3] *Hind Ahmed Gasimelseed,*[4] *Minas M. Balla,*[5] *Amani M.A Bakhiet,*[6] *Khalid Berma,* [7] *Mohamed Alfaki,*[8,*] *and Maria V. Babak*[1,*]

[1] Drug Discovery Lab, Department of Chemistry, City University of Hong Kong, 83 Tat Chee Avenue, Hong Kong SAR, 999077, People's Republic of China

[2] Department of Biology and Biochemistry, Faculty of Sciences I, Lebanese University, Old Saida Rd, Hadath, Beirut, Lebanon

[3] Faculty of Pharmacy, International University of Africa, Madani St., Khartoum, Sudan

[4] Department of Immunology and Biotechnology, Tropical Medicine Research Institute, National Center for Research, El Mek Nimir Avenue, Khartoum, Sudan

[5] Faculty of Sciences, University of Gezira, University Avenue, Wad Madani, Sudan

[6] College of Science, Hafr Al-Batin University, King Abdulaziz Rd, Hafar Al-Batin, Eastern, Saudi Arabia

[7] Department of Biotechnology, Faculty of Science and Technology, Omdurman Islamic University, Salha St., Khartoum, Sudan

[8] Software Engineering Department, Faculty of Computer Science, Al-Neelain University, El Gamhuriya Avenue, Khartoum, Sudan

[†] These authors contributed equally to this work

*Correspondence: Mohamed.alkhair.alfaki@gmail.com (MA), mbabak@cityu.edu.hk (MVB)

Keywords: PAPPA, pan-cancer, tumor microenvironment, cancer-associated fibroblasts, promoter methylation, prognostic biomarker

Table of contents

Table of contents 2
Supplementary Methods 3
Bioinformatic resources and database specifications 3
Processing of microarray validation datasets 3
Immunohistochemical (IHC) staining evaluation 4
Survival analysis and statistical parameters 4
Functional enrichment and network construction 5
Supplementary Tables 6
Supplementary Table S1. Modules used in each database and the query parameters applied in each analysis, and the access periods. 6
Supplementary Table S2. Cancer types and the expression profile compared to normal tissue in GEPIA2, TIMER2.0, and UALCAN. 7
Supplementary Table S3: Correlation between PAPPA expression and immune cell infiltration across selected cancers using TIMER2.0. 8
Supplementary Table S4: Overview of PAPPA tumor-specific characteristics across selected cancers. 9
Supplementary figures 10
Supplementary Figure S1: PAPPA single-cell expression analysis 10
Supplementary Figure S1 (continued): PAPPA single-cell expression analysis. 11
Supplementary Figure S2: Clinical parameters of PAPPA expression across cancer types analyzed using the UALCAN database 12
Supplementary Figure S3: PAPPA promoter DNA methylation and corresponding gene expression analysed in dysregulated cancers. 13
Supplementary Figure S4: Scatter plots for sample-level correlations between PAPPA promoter methylation and gene expression across TCGA cohorts using Spearman correlation. 14
Supplementary Figure S5: Kaplan-Meier curves for Overall Survival (OS) based on PAPPA expression levels. 15
Supplementary Figure S6: PAPPA gene expression level correlation with the immune cells infiltration level in different cancer types using the TIMER database 16

Supplementary Figure S6 (continued): PAPPA gene expression level correlation with the immune cells infiltration level in different cancer types using the TIMER database. 17

Supplementary Figure S7: Overall survival (OS) and disease-free survival (DFS) between genetically altered and unaltered PAPPA groups. 18

Supplementary Methods

Bioinformatic resources and database specifications

Initial gene expression screening and immunological analyses were conducted using the TIMER2.0 platform (https://compbio.cn/timer2/, accessed in February 2025), GEPIA (http://gepia.cancer-pku.cn/, accessed in February 2025), and UALCAN (https://ualcan.path.uab.edu/, accessed in February 2025), which provided preliminary data on tumor-normal differential expression and promoter methylation status. To assess expression at single-cell resolution, we utilized the TISCH2 database (http://tisch.comp-genomics.org/, accessed in February 2025), evaluating log-normalized expression values (logTPM) across pre-defined cellular clusters. Genetic alterations, including somatic mutations and copy number variations, were investigated using the cBioPortal for Cancer Genomics (https://www.cbioportal.org/, accessed in February 2025), leveraging data from the TCGA Pan-Cancer Atlas to identify potential genomic drivers associated with PAPPA dysregulation. Cox risk regression analysis was performed using TIMER2.0 (https://compbio.cn/timer2/, accessed January 2026). The modules and query parameters used, along with the analysis performed on each database, are represented in Supplementary Table 1.

Processing of microarray validation datasets

For external validation, raw gene expression datasets were retrieved from the Gene Expression Omnibus (GEO). Data processing was performed within the R statistical environment (version 4.5.0; R Foundation for Statistical Computing, Vienna, Austria). Where necessary, raw intensity values were log2-transformed (log2(x+1)) to ensure normal distribution. Probe identifiers were mapped to Human

Genome Organisation (HUGO) Gene Nomenclature Committee (HGNC) symbols using the appropriate Bioconductor annotation packages. In cases where multiple probes mapped to a single gene, expression values were averaged to obtain a unique gene-level value. Differential expression analysis was subsequently performed using the linear models for microarray data (limma) package. For unpaired study designs, a design matrix was constructed without an intercept, while paired samples included patient identity as a blocking factor. Variances were moderated using the empirical Bayes procedure, and statistical significance was defined using a Benjamini-Hochberg False Discovery Rate (FDR) threshold of < 0.05.

Immunohistochemical (IHC) staining evaluation

To validate transcriptomic findings at the proteomic level, immunohistochemical staining patterns were analyzed using high-resolution images from the Human Protein Atlas (HPA). The specific antibody analyzed was the Sigma-Aldrich HPA001667. Staining patterns were manually evaluated by a certified pathologist to determine cellular localization (cytoplasmic versus membranous) and staining intensity. Intensity was semi-quantitatively scored as negative, weak, moderate, or strong, and specificity was confirmed by comparing staining patterns in tumor cells against the surrounding stromal and normal tissue components.

Survival analysis and statistical parameters

Prognostic evaluation was performed using both web-based tools (GEPIA2, Kaplan-Meier Plotter, and TISCH2) and manual curation of TCGA/TARGET clinical data. Survival analyses reported via TISCH2 are derived from TCGA datasets, rather than from single-cell sequencing samples themselves. Patients were stratified into high- and low-expression cohorts using the median gene expression value as the cutoff threshold unless an optimal cutoff was otherwise specified by the database algorithm. Survival probability was estimated using the Kaplan-Meier method, with differences between groups assessed

via the log-rank test. Hazard ratios (HR) and 95% confidence intervals (CI) were calculated using univariate Cox proportional hazards regression models. All statistical tests were two-sided, and a P-value < 0.05 was considered statistically significant. Cohort size, number of events, and follow-up durations for GEPIA and KM-Plotter correspond to datasets utilized by each platform and are reported within respective portal outputs.

Functional enrichment and network construction

Protein-protein interaction (PPI) networks were constructed by integrating data from STRING (https://string-db.org/, accessed in February 2025) and GeneMANIA (http://genemania.org/, accessed in February 2025). The top 10 interacting genes from each platform were intersected to define a high-confidence gene set. This gene set was subsequently subjected to Gene Ontology (GO) and Kyoto Encyclopedia of Genes and Genomes (KEGG) pathway enrichment analyses using the Enrichr platform (https://maayanlab.cloud/Enrichr/, accessed in February 2025). Enrichment significance was determined using Fisher's exact test, with Benjamini-Hochberg-adjusted P-values provided by Enrichr.Terms with an adjusted P-value < 0.05 were considered statistically significant.

Supplementary Tables

Supplementary Table S1. Modules used in each database and the query parameters applied in each analysis, and the access periods.

Resource	Access Date	Analysis	Query Parameters
**TIMER**	Feb, 2025	Immune Infiltration	"Gene" module: Input gene → Select 6 default immune cells
**TIMER2.0**	Feb, 2025	Gene Expression	"Gene_DE" module: Input Gene
	Feb, 2025	Gene-Gene correlation in cancer	"Gene_Corr" Module: Input Gene → Input List of Possible Correlated Genes→ Enable Purity-Adjustment
	Feb, 2025	Gene Expression correlation with CAF Infiltration	Immune Module: Input Gene → Input immune infiltrate (CAF) → Enable Purity-Adjustment
	Jan, 2026	Cox Risk Regression Analysis	Outcome Module: Immune infiltrate (CAF) → Clinical (purity) → Gene expression (Input Gene)
**GEPIA2**	Feb, 2025	Gene Expression	"Expression DIY": Box Plot → Input Gene → |log2FC| > 1.5, p-value < 0.05, log scale: yes → Select Datasets (TCGA/GTEx)
		Survival Analysis	"Survival Analysis" → Input Gene → Overall Survival → Group Cutoff (Median) → Enable HR & 95% CI
**UALCAN**	Feb, 2025	Gene Expression	"TCGA Gene": Input Gene → Select Cancer Type → Expression link
		DNA Methylation	"TCGA Gene": Input Gene → Select Cancer Type → Methylation link
**TISCH2**	Feb, 2025	Gene Expression	"Gene": Input Gene → Select Cancer Type → Average gene expression
		Survival Analysis	"Gene": Input Gene → Select Cancer Type → TCGA Survival
**cBioPortal**	Feb, 2025	Genomic Alterations	Select TCGA PanCancer Atlas Studies → Query by Gene → "Cancer Type Summary"/ "Mutations"
		Survival Analysis	Select TCGA PanCancer Atlas Studies → Query by Gene → "Comparison/Survival" → Survival tab
	Jan, 2026	Sample level Expression-Methylation Correlation	Select Study: "Cancer Type (TCGA, PanCancer Atlas)" → Query by Gene → "Plots"
**KM-Plotter**	Feb, 2025	Gene Expression/OS	Pan-cancer RNA-seq: Input Gene → Auto-select best cutoff

Supplementary Table S2. Cancer types and the expression profile compared to normal tissue in GEPIA2, TIMER2.0, and UALCAN. D: downregulated, U: upregulated, ND: No Data, NND: No Normal tissue Data, *: P < 0.05, **: P < 0.01, ***: P < 0.001.

Cancer Type		GEPIA2	TIMER2.0	UALCAN
ACC	Adrenocortical carcinoma	D	NND	ND
BLCA	Bladder Urothelial Carcinoma	D	D*	D
BRCA	Breast invasive carcinoma	D	D***	D***
CESC	Cervical squamous cell carcinoma and endocervical adenocarcinoma	D***	D*	D
CHOL	Cholangiocarcinoma	U	U***	U***
COAD	Colon adenocarcinoma	D	D*	D
DLBC	Lymphoid Neoplasm Diffuse Large B-cell Lymphoma	U	NND	ND
ESCA	Esophageal carcinoma	U	U	U*
GBM	Glioblastoma multiforme	U	U	U
HNSC	Head and Neck squamous cell carcinoma	U	U*	U
KICH	Kidney Chromophobe	D***	D***	D***
KIRC	Kidney renal clear cell carcinoma	D***	D***	D***
KIRP	Kidney renal papillary cell carcinoma	D	D***	D*
LAML	Acute Myeloid Leukemia	D	NND	ND
LGG	Brain Lower Grade Glioma	U	NND	ND
LIHC	Liver hepatocellular carcinoma	D	D	D
LUAD	Lung adenocarcinoma	D	No change	D*
LUSC	Lung squamous cell carcinoma	D	D	D
MESO	Mesothelioma	ND	NND	ND
OV	Ovarian serous cystadenocarcinoma	D	NND	ND
PAAD	Pancreatic adenocarcinoma	U	D	D
PCPG	Pheochromocytoma and Paraganglioma	D	D	D
PRAD	Prostate adenocarcinoma	D	D***	D***
READ	Rectum adenocarcinoma	D	D	D
SARC	Sarcoma	D	NND	D
SKCM	Skin Cutaneous Melanoma	D	NND	D
STAD	Stomach adenocarcinoma	U	U	U*
TGCT	Testicular Germ Cell Tumors	D	NND	ND
THCA	Thyroid carcinoma	D	U***	U***
THYM	Thymoma	U	NND	D
UCEC	Uterine Corpus Endometrial Carcinoma	D***	D***	D***
UCS	Uterine Carcinosarcoma	D***	NND	ND
UVM	Uveal Melanoma	ND	NND	ND

Supplementary Table S3: Correlation between PAPPA expression and immune cell infiltration across selected cancers using TIMER2.0. Partial correlation coefficients (r) and corresponding *P* values are provided for tumor purity, B cells, $CD8^+$ T cells, $CD4^+$ T cells, macrophages, neutrophils, and dendritic cells.

Cancer	Immune Infiltration													
	Purity		B cells		CD8+		CD4+		Macrophage		Neutrophil		Dendritic cells (DC)	
	Partial cor	P value	Partial cor	P value	Partial cor	P value	Partial cor	P value	Partial cor	P value	Partial cor	P value	Partial cor	P value
**BLCA**	–0.401	1.09E-15	–0.167	0.001	0.278	6.45E-08	0.15	0.004	0.111	0.034	0.344	1.66E-11	0.376	1.00E-13
**CESC**	–0.194	0.001	–0.073	0.224	–0.005	0.931	–0.173	0.004	0.013	0.829	–0.011	0.854	0.051	0.396
**LGG**	0.131	0.004	–0.239	1.17E-07	–0.028	0.547	–0.139	0.002	–0.097	0.034	–0.179	8.98E-05	–0.207	5.26E-06
**LUSC**	–0.308	5.72E-12	–0.019	0.683	0.105	0.022	0.243	8.08E-08	0.217	1.79E-06	0.301	2.16E-11	0.252	2.74E-08
**MESO**	–0.189	0.081	0.124	0.261	0.263	0.016	–0.087	0.43	0.105	0.344	–0.139	0.206	0.291	0.007
**PAAD**	–0.104	0.176	0.323	1.7E-05	0.512	8.57E-13	0.066	0.391	0.564	9.51E-16	0.506	1.67E-12	0.558	2.20E-15
**STAD**	–0.276	4.77E-08	0.142	0.006	0.026	0.614	0.177	0.00065	0.252	9.08E-07	0.189	0.000243	0.129	0.013

Supplementary Table S4: Overview of PAPPA tumor-specific characteristics across selected cancers. Summary of PAPPA tumor-specific features, including the putative cellular source of expression (based on single-cell RNA-seq analysis), the direction of correlation between PAPPA expression and cancer-associated fibroblast (CAF) infiltration, and the overall survival (OS) association.

Tumor Type	Cell Origin	CAF Correlation	OS Direction
BLCA	Fibroblast	Positive	Risk factor
CESC	Fibroblast	Positive	Risk factor
LUSC	Fibroblast	Positive	Risk factor
MESO	NA	Positive	Risk factor
PAAD	Fibroblast	Positive	Risk factor
STAD	Fibroblast	Positive	Risk factor
LGG	NA	Negative	Protective factor

Supplementary figures

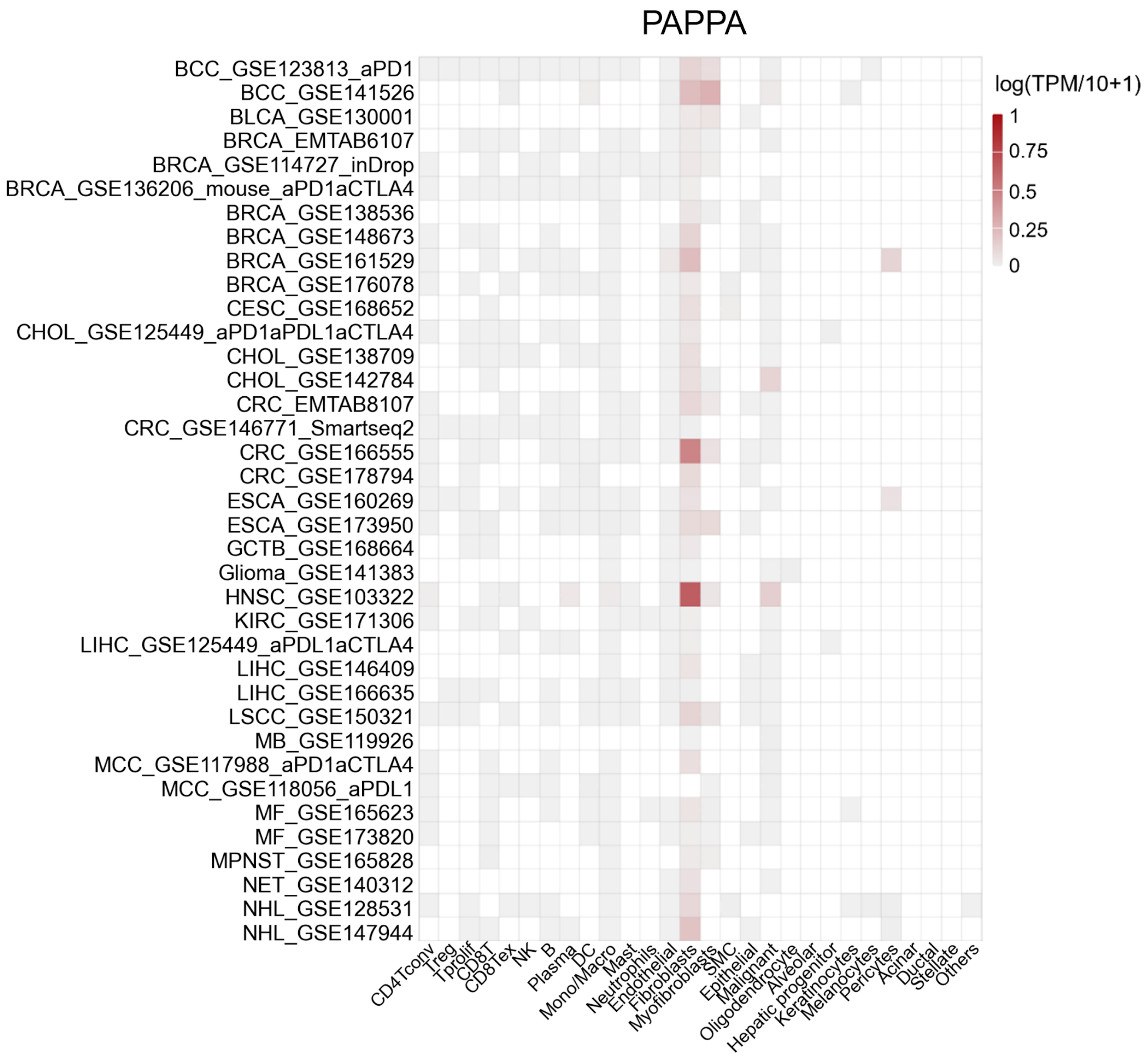

Supplementary Figure S1: PAPPA single-cell expression analysis (continued on the next page). Heatmap of *PAPPA* scRNA-seq expression across cancer types from the TISCH2 database.

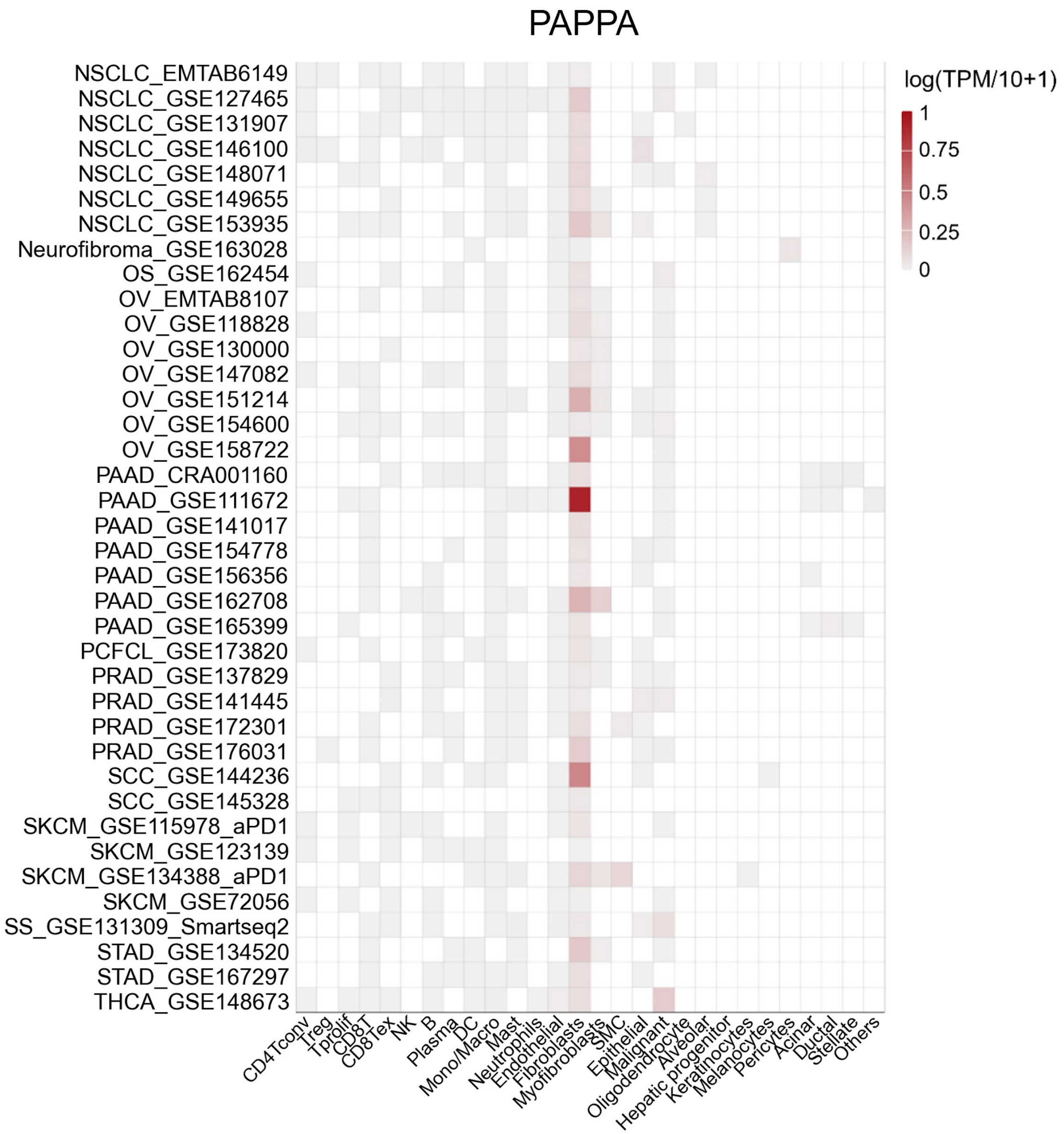

Supplementary Figure S1: PAPPA single-cell expression analysis. Heatmap of PAPPA scRNA-seq expression across cancer types from the TISCH2 database.

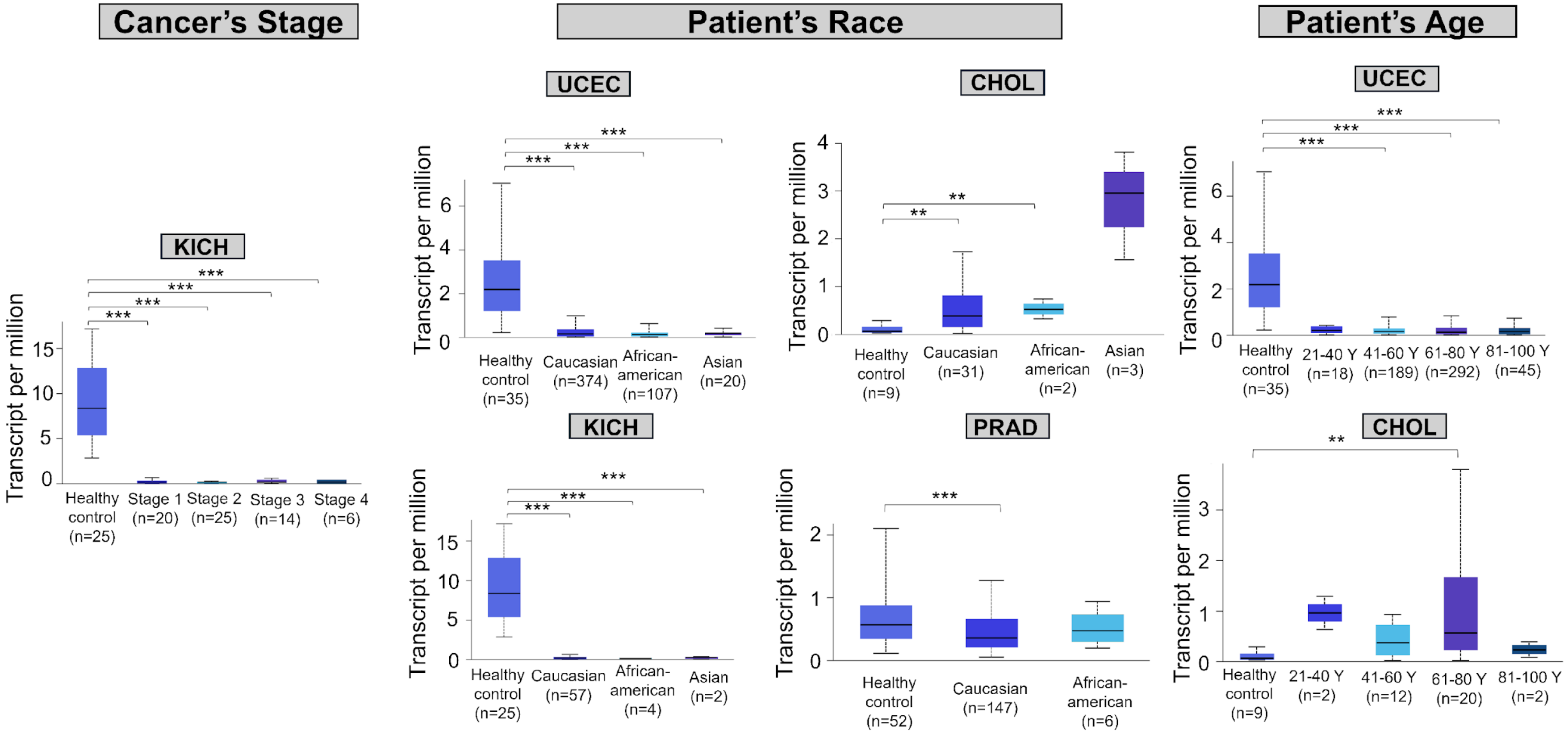

Supplementary Figure S2: Clinical parameters of PAPPA expression across cancer types analyzed using the UALCAN database. Box plots depict PAPPA expression levels (transcripts per million, TPM) in normal versus tumor tissues, stratified by cancer stage, race, and age. Sample sizes for each group (n) are indicated. Statistical significance is denoted by asterisks (*: $P < 0.05$; **: $P < 0.01$; ***: $P < 0.001$).

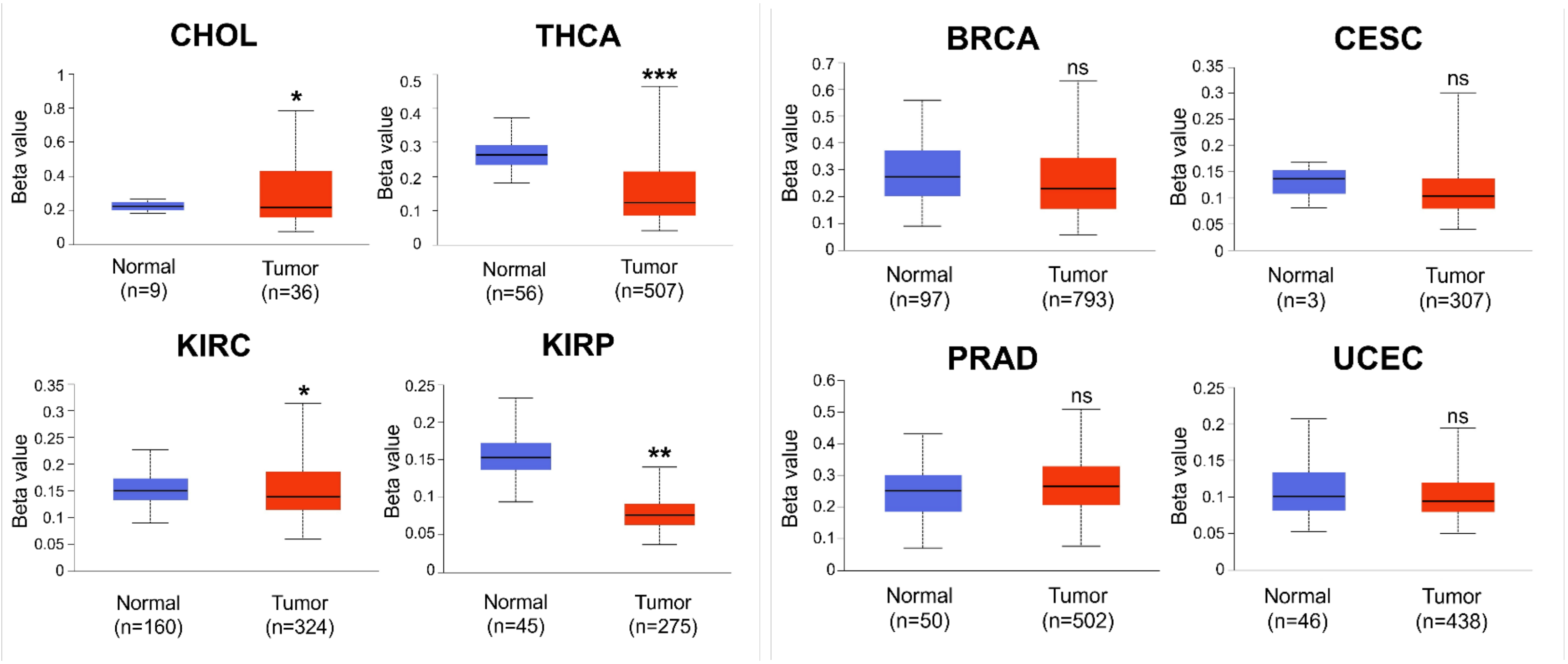

Supplementary Figure S3: PAPPA promoter DNA methylation and corresponding gene expression analysed in dysregulated cancers. Box plots display β values representing promoter methylation levels and transcripts per million representing PAPPA mRNA expression. Blue boxes indicate normal tissue samples, and red boxes indicate primary tumor samples. Sample sizes (n) are shown for each group. Statistical significance is annotated by asterisks (*: $p < 0.05$; **: $p < 0.01$; ***: $p < 0.001$).

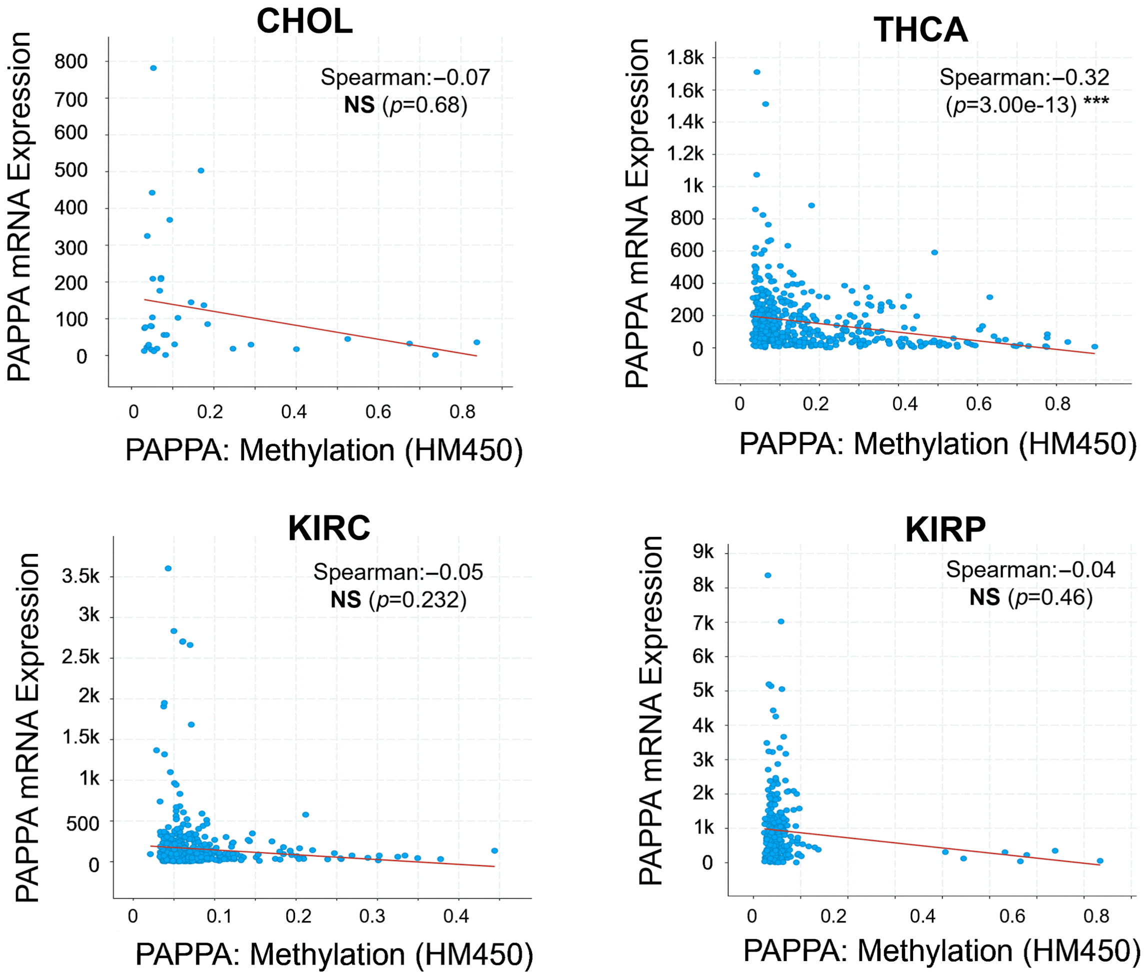

Supplementary Figure S4: Scatter plots for sample-level correlations between PAPPA promoter methylation and gene expression across TCGA cohorts using Spearman correlation. Scatter plots showing Spearman correlations between PAPPA promoter methylation levels and PAPPA gene expression across TCGA tumor cohorts. Each dot represents an individual tumor sample. Cohort sizes: CHOL (n = 36), THCA (n = 496), KIRC (n = 512), and KIRP (n = 283). Statistical significance is indicated by asterisks (*$P < 0.05$; **$P < 0.01$; ***$P < 0.001$).

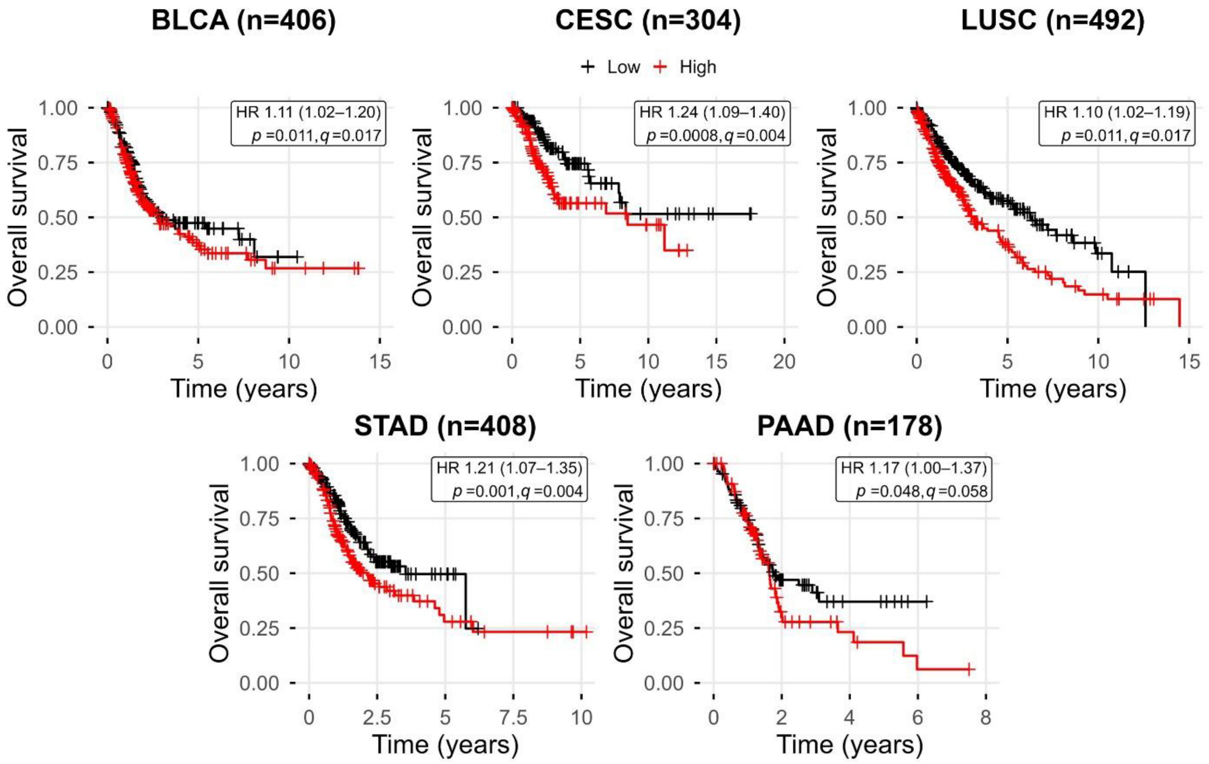

Supplementary Figure S5: Kaplan-Meier curves for Overall Survival (OS) based on PAPPA expression levels. Patients were stratified into high-expression (red) and low-expression (black) groups based on the median PAPPA expression (median split). Hazard Ratios (HR) and 95% Confidence Intervals (CI) were calculated using univariate Cox regression models. P-values were adjusted for multiple testing using the Benjamini-Hochberg (FDR) method.

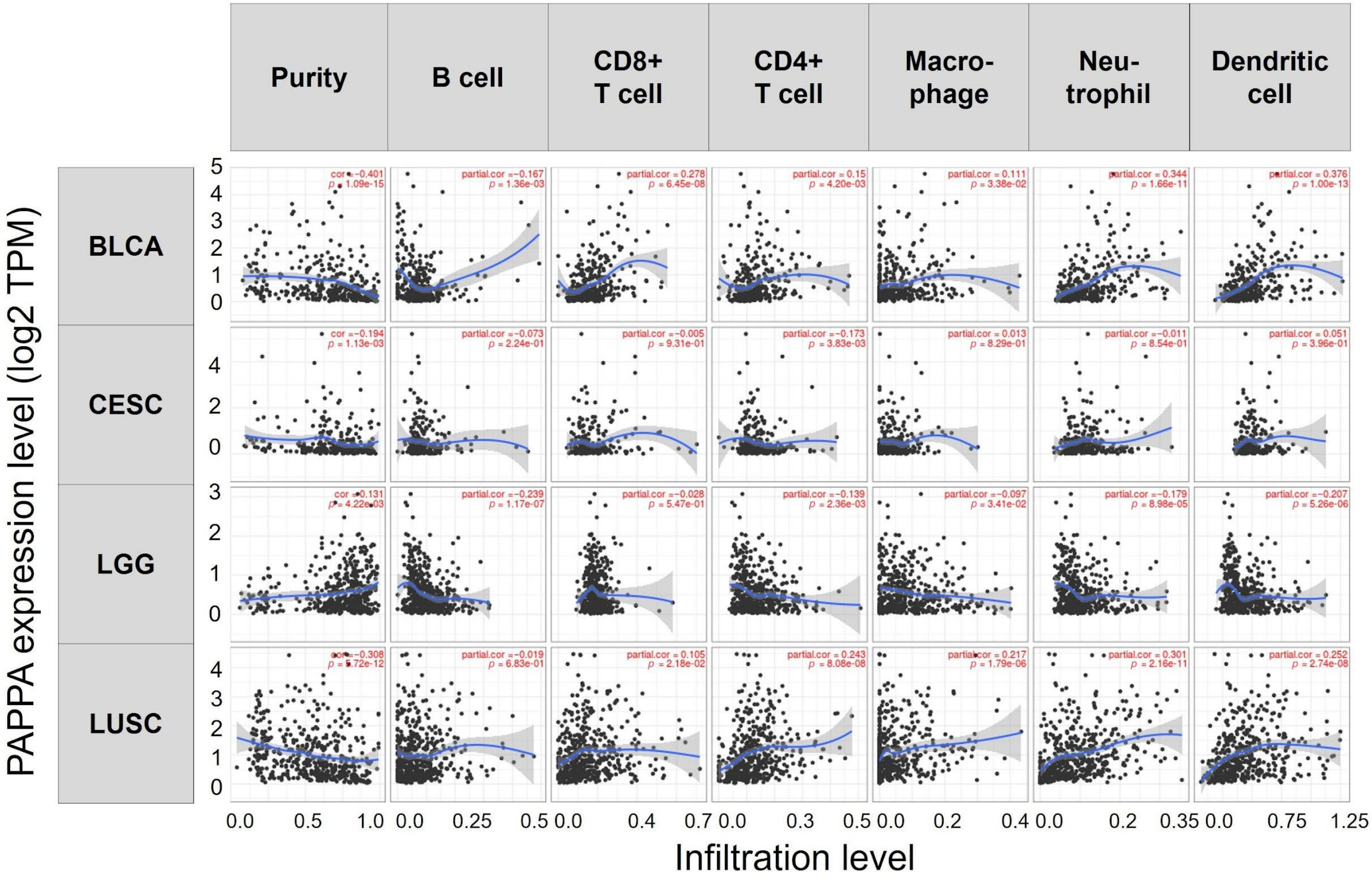

Supplementary Figure S6: PAPPA gene expression level correlation with the immune cells infiltration level in different cancer types using the TIMER database (continued on the next page). Scatter plots show *PAPPA* mRNA levels ($\log_2$ TPM) versus estimated

infiltration levels of tumor purity, B cells, CD8⁺ T cells, CD4⁺ T cells, macrophages, neutrophils and dendritic cells in BLCA, CESC, LGG, LUSC, MESO, PAAD and STAD. Each point represents an individual sample; blue curves indicate fitted regression lines with 95% confidence intervals (grey shading). Partial correlation coefficients and corresponding p-values from TIMER are shown in red in each panel.

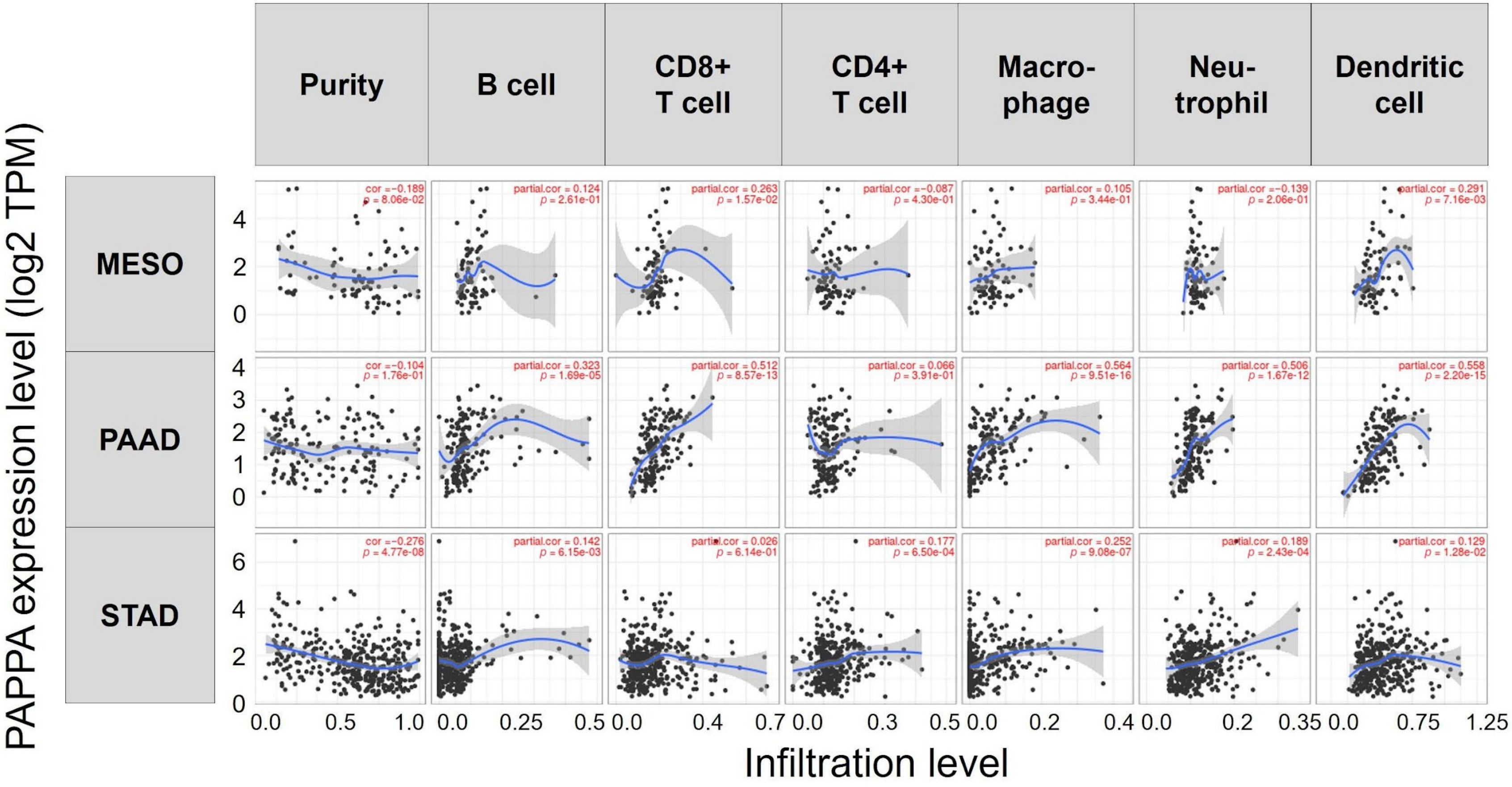

Supplementary Figure S6: PAPPA gene expression level correlation with the immune cells infiltration level in different cancer types using the TIMER database. Scatter plots show *PAPPA* mRNA levels (log_2 TPM) versus estimated infiltration levels of tumor

purity, B cells, CD8+ T cells, CD4+ T cells, macrophages, neutrophils and dendritic cells in BLCA, CESC, LGG, LUSC, MESO, PAAD and STAD. Each point represents an individual sample; blue curves indicate fitted regression lines with 95% confidence intervals (grey shading). Partial correlation coefficients and corresponding p-values from TIMER are shown in red in each panel.

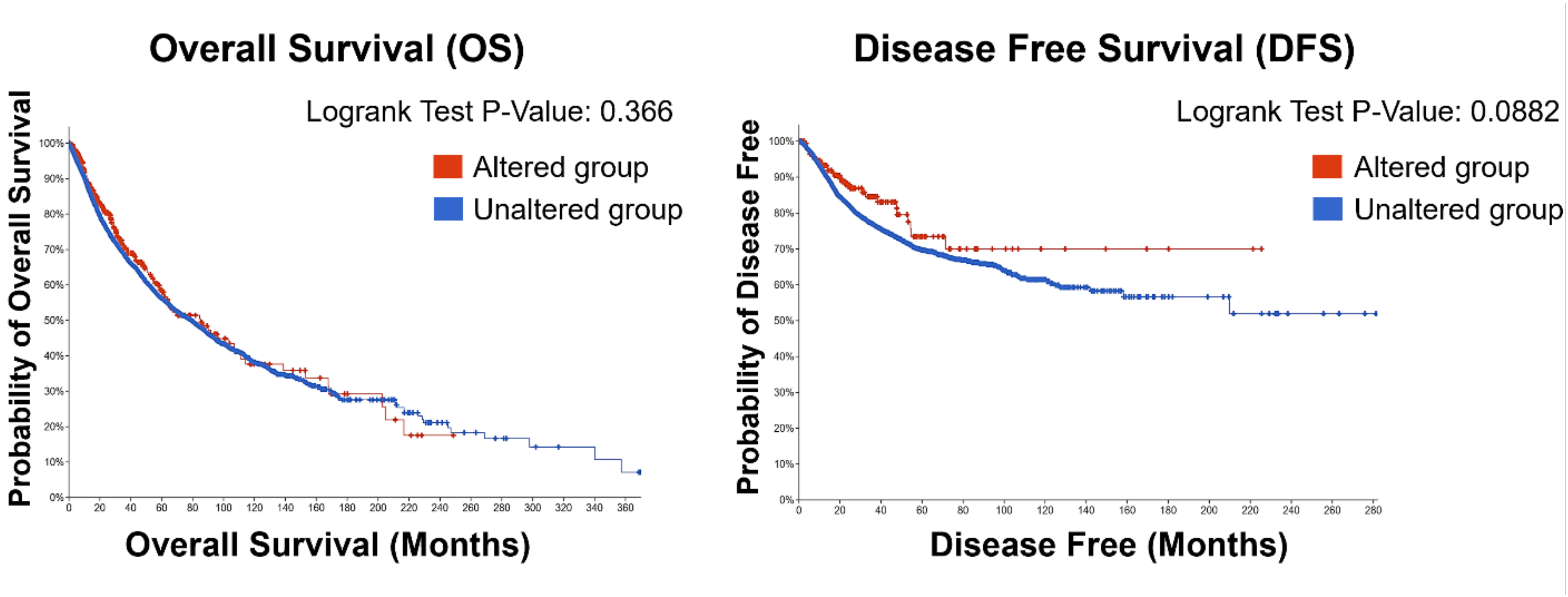

Supplementary Figure S7: Overall survival (OS) and disease-free survival (DFS) between genetically altered and unaltered PAPPA groups. Correlation between alterations in *PAPPA* and overall survival (OS) and disease-free survival (DFS) using the cBioPortal database.
